# Auto-branch multi-task learning for simultaneous prediction of multiple correlated traits associated with Alzheimer’s disease

**DOI:** 10.3389/fgene.2025.1538544

**Published:** 2025-06-10

**Authors:** Jiaqi Liang, Zhao Xue, Wenchao Zhou, Xiangjie Guo, Yalu Wen

**Affiliations:** ^1^ Academy of Medical Sciences, Shanxi Medical University, Taiyuan, Shanxi, China; ^2^ Department of Health Statistics, School of Public Health, Shanxi Medical University, Taiyuan, Shanxi, China; ^3^ Translational Medicine Research Center, Shanxi Medical University, Taiyuan, Shanxi, China; ^4^ School of Forensic Medicine, Shanxi Medical University, Jinzhong, Shanxi, China; ^5^ Department of Statistics, University of Auckland, Auckland, New Zealand

**Keywords:** alzheimer’s disease, multi-task learning, phenotype prediction, deep learning, autobranch method, genetic analysis

## Abstract

**Introduction:**

Correlated phenotypes may have both shared and unique causal factors, and jointly modeling these phenotypes can enhance prediction performance by enabling efficient information transfer.

**Methods:**

We propose an auto-branch multi-task learning model within a deep learning framework for the simultaneous prediction of multiple correlated phenotypes. This model dynamically branches from a hard parameter sharing structure to prevent negative information transfer, ensuring that parameter sharing among phenotypes is beneficial.

**Results:**

Through simulation studies and analysis of seven Alzheimer's disease-related phenotypes, our method consistently outperformed Multi-Lasso model, single-task learning approaches, and commonly used hard parameter sharing models with predefine shared layers. These analyses also reveal that while genetic contributions across phenotypes are similar, the relative influence of each genetic factor varies substantially among phenotypes.

## 1 Introduction

Alzheimer’s disease (AD) is a progressive neurodegenerative disorder, with its prevalence increasing annually (AuthorAnonymous, 2023). Approximately 50 million individuals worldwide are affected by dementia with approximately 60%–70% being AD cases, and this figure is projected to rise to 152 million by 2050 (Santiago et al., 2019; Zhang et al., 2021). AD is a multifactorial condition manifested through various traits, such as cognitive decline and functional changes (Löffler et al., 2014; Jabir et al., 2021). Genetic risk prediction models have been developed for various AD-related traits, but these models usually only focus on one trait, ignoring their inter-relationships (Jung et al., 2020; Zhu et al., 2024). Although each AD-related trait provides valuable information on the genetic risk of AD, none of them alone can capture the full complexity of the disease and a comprehensive model that can jointly model multiple traits is needed.

Cognitive and functional changes commonly observed in AD patients can be assessed using several tools, including the Mini-Mental State Examination (MMSE), Montreal Cognitive Assessment (MoCA), Clinical Dementia Rating-Sum of Boxes (CDRSB), Alzheimer’s Disease Assessment Scale-Cognitive Subscale 13 (ADAS13), and the Functional Activities Questionnaire (FAQ). MMSE and MoCA assess general cognitive impairment, with MoCA being more sensitive in detecting early AD (Pinto et al., 2019; Duc et al., 2020). ADAS13 and CDRSB are designed for tracking AD progression. However, ADAS13 measures the severity of cognitive symptoms (Bucholc et al., 2019), whereas CDRSB assesses both cognitive and functional domains, offering a more comprehensive view of how AD affects a patient’s daily life (Cullen et al., 2020). FAQ focuses on assessing functional ability in daily activities (Petersen et al., 2021). Neuroimaging is also used in AD diagnosis and monitoring (Besson et al., 2015; Winer et al., 2018). For example, florbetapir (AV45) detects the amyloid-beta plaque in the brain (Mattson, 2004; Johnson et al., 2013). Fluorodeoxyglucose (FDG) measures brain glucose metabolism and identifies regions of hypometabolism (de Paula Faria et al., 2022). While AD assessment tools provide valuable information, none of them alone can be treated as a gold standard for AD diagnosis, especially for early-stage cases. For example, although amyloid plaque is a hallmark feature of AD, some individuals with such manifestations never develop into AD (Reinitz et al., 2022).

PET-imaging, cognitive, and functional changes provide confirmatory and complementary information regarding AD risk. Simultaneous modelling of them can leverage information across traits, which facilitates the detection of new biomarkers and improves the overall prediction. However, existing prediction models mainly focus on a single trait. For example, traditional models such as gBLUP build separate prediction models for each trait (de Los Campos et al., 2013). This trait-specific focus persists even within the deep learning domain. For example, Duc et al. (2020) employed a single task learning (STL) model to automatically diagnose AD (Duc et al., 2020). Liu et al. (2022) introduced an interpretable STL model to assess the risk of AD based on high-dimensional genomic data, where PET imaging outcomes were predicted separately (Liu et al., 2022). Notably, some studies have explored modeling multiple tasks simultaneously. For example, a classic multi-task model, Multi-Lasso, has shown promising performance when applied on SNPs data (Wang et al., 2012; Bee et al., 2024). This method, originally proposed by Obozinski et al. (2006), applies joint sparse regularization through *ℓ*_2_,_1_-norm across tasks, enabling feature sharing among related tasks. It has been applied in several studies (Wang et al., 2012; Bee et al., 2024), including the recent application on Alzheimer’s Disease Neuroimaging Initiative (ADNI) dataset (Cheng et al., 2019). These studies indicate the potential of multi-task modeling strategies in the context of AD prediction, although their practical application remains limited.

In recent years, Multi-task learning (MTL) has been widely applied in the field of deep learning as an effective strategy to improve model performance. It has been successfully applied to model multiple correlated outcomes, particularly in typical deep learning scenarios such as natural language processing (Zhang et al., 2020; Li et al., 2022) and image classification (Liu et al., 2021). Current deep MTL approaches can be broadly categorized into hard and soft parameter sharing models. Hard parameter sharing models use shared layers across tasks, typically sharing all layers except the last to learn a common representation while capturing task-specific characteristics (Vandenhende et al., 2022). Recent advances enable these models to automatically determine which layers to share. For example, the Fully Adaptive Feature Sharing method dynamically widens layers based on similarities among tasks (Lu et al., 2017). The Multilinear Relationship Network discovers inter-task relationships and alleviates the dilemma of negative transfer by jointly training transferable features (Long et al., 2017). The Task Affinity Grouping optimizes layer sharing by branching the network according to inter-task affinity scores (Fifty et al., 2021). Unlike hard sharing, soft parameter sharing models allow each task to maintain independent parameters and control the levels of sharedness using additional parameters. They offer greater flexibility, but at the cost of increased computational demands. The cross-stitch network represents a classic example, where additional parameters are introduced in the cross-stitch unit to ascertain the optimal degree of sharedness (Misra et al., 2016). AD-related traits, such as cognitive scores, functional assessments, and neuroimaging findings are interconnected. MTL approaches, especially those computationally efficient hard parameter sharing models, have great potential to enhance generalization, learning efficiency and overall prediction accuracy for genetic risk predictions. However, existing MTLs have been rarely used in such applications, partially due to the low signal-to-noise ratio and unclear levels of genetic relatedness.

We here developed an auto-branch multi-task learning model for the prediction analyses of multiple correlated traits using genetic data. Our method can distinguish and integrate commonalities and unique characteristics across multiple traits, leading to improved prediction performance, as measured by both Pearson correlation and root mean squared error (RMSE). In the following sections, we first provided the technical details of our method and then conducted extensive simulation studies to evaluate its performance. Finally, we built genetic risk prediction models for multiple AD-related traits, including cognitive and functional assessments and PET imaging outcomes, using data sourced from the ADNI (Mueller et al., 2005).

## 2 Methods

**FIGURE 1:**
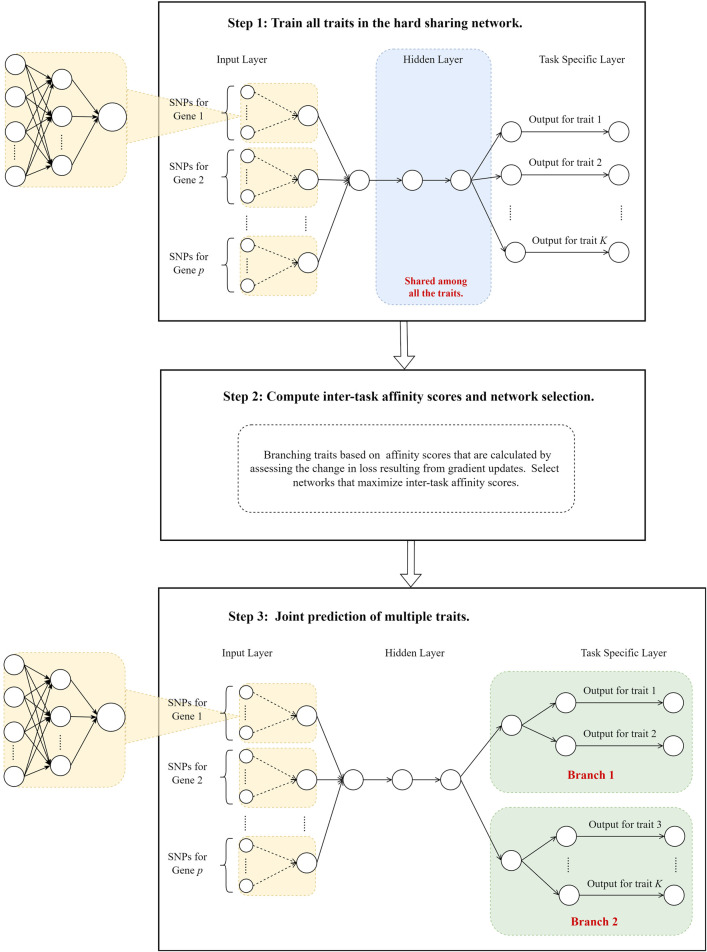
The overview of the study design.

Our method is developed using the idea originally proposed by Fifty et al. (2021) in the analyses of facial image dataset CelebA (Liu et al., 2015) and computer vision dataset Taskonomy (Zamir et al., 2018). In hard parameter sharing models, the gradient update of one task can influence others. If the gradient update of one task reduces the loss of another, a “synergistic effect” between the two tasks is observed. Conversely, an “antagonistic effect” occurs when the update negatively impacts the other task. Jointly training can enhance model performance for synergistic tasks by leveraging their positive correlations, but it may reduce performance for antagonistic tasks. We proposed to quantify the “synergistic effect” among correlated traits and branch the network where traits are considered antagonistic. Specifically, we first constructed a hard parameter sharing model with all layers except the last shared to predict multiple traits using genetic data. We then quantified trait similarities and grouped traits using the inter-trait affinity (Fifty et al., 2021). Finally, we branched the hard parameter sharing model for traits that are deemed antagonistic. Unlike Fifty et al. (2021), who used separate models for traits in each group, we proposed to use a hard-parameter sharing strategy and branch the network when phenotypes are “antagonistic.” This is mainly because correlated phenotypes are likely to have shared genetic determinants (Badré and Pan, 2023), and we hypothesized that this can be exploited to improve predictions. The overview of our workflow is in Figure 1.

### 2.1 Train all traits together in a hard parameter sharing model

We proposed to use a hard parameter sharing model, where all layers except for the last one are shared, to train prediction models for all traits and evaluate the effect of gradient update from one task on another. We utilized the shared layers to capture the common representations among these traits and used the last trait-specific layer to handle their uniqueness. To consider biological information and improve model interpretation, we added a customized layer right after the input layer (Step 1 in Figure 1), where predictors from the same genes are first grouped together and then fed to the downstream networks. This customized layer acts similarly to those set-based analyses (Wang et al., 2016; Chen et al., 2019), where weak signals within a gene are aggregated, enhancing the overall performance of the models. Note that although we aggregated the signals within each gene, a similar layer can be designed based on other biological information (e.g., pathways). As opposed to image classification, the signals in genetic data are weak and can lead to poor prediction models without signal enhancement, which can have a profound impact on gauging the trait similarities.

### 2.2 Branch network based on inter-trait affinity

Within the hard parameter-sharing framework, traits transfer information to each other through successive gradient updates to the shared parameters. For traits that are intrinsically similar, the update in one trait’s gradient on the shared parameters would lead to a reduction in loss for the others. On the contrary, this update can lead to a negligible or an increase in loss for independent traits. Therefore, we used inter-task affinity scores, calculated based on loss changes during parameter training (Fifty et al., 2021), to evaluate the pairwise similarity among traits. These scores are further used to determine whether traits should be trained together or branched. Specifically, we gathered gradient information during the training of the model outlined in session 2.1. We calculated the pairwise trait affinity during each parameter update, where the affinity was defined as the extent to which the gradient update of shared parameters by trait *i* impacted the loss of trait *j* (Fifty et al., 2021). At step *t*, following a gradient update of the shared parameters based on the loss of trait *i*, the affinity of trait *i* to trait *j* is calculated as shown in Equation 1:

$$Z_{i\to j}^{t}=1-\frac{L_{i\to j}^{t+1}}{L_{i\to j}^{t}},$$ (1)

where $L_{i\to j}^{t}$ and $L_{i\to j}^{t+1}$ respectively denote the loss of trait *j* before and after shared parameter updates based on task *i* at step *t*. If the affinity is greater than 0, it signifies that the gradient update of task *i* positively influences trait *j*. We defined the overall affinity between trait *i* and trait *j* as an average over *T* epochs, as shown in Equation 2:

$${\hat{Z}}_{i\to j}=\frac{1}{T}\sum_{t=1}^{T}Z_{i\to j}^{t}.$$ (2)

To enable efficient information transfer, traits within the same branch are expected to have high pairwise affinities ( ${\hat{Z}}_{i\to j}$ ). Let $S_{i}$ be the set of traits that are in the same branch as trait *i* and $\left|S_{i}\right|$ be the cardinality of the set. The inter-trait affinity for trait *i* can determine the synergistic effects among all traits in the same branch (i.e., set $S_{i}$ ) and is defined in Equation 3:

$${\hat{Z}}_{i}=\frac{\sum_{j\in S_{i}}{\hat{Z}}_{j\to i}}{\left|S_{i}\right|}.$$ (3)

Obviously, ${\hat{Z}}_{i}$ is large when all traits in the branch are similar to trait *i* (i.e., ${\hat{Z}}_{j\to i}$ is large), and it is small when traits are independent. We define the total inter-trait affinity for *K* traits as shown in Equation 4:

$$Z=\frac{1}{K}\sum_{i}{\hat{Z}}_{i}.$$ (4)

Identifying the best parameter sharing (i.e., branching) strategy is equivalent to finding the optimal number of branches and the partition of the traits that maximize the total inter-trait affinity defined in Equation 4. However, this problem is an NP-hard problem (Fifty et al., 2021). From the practical perspective, we treat the optimal number of branches as a prior and find the best partition of traits that maximize Z .

### 2.3 Joint prediction of multiple traits

Given a pre-specified number of branches and its corresponding best partition of traits, we constructed a hard parameter sharing model with traits in different partitions branched (Step 3 in Figure 1). As phenotypes are likely to have shared genetic determinants, we set the top few hidden layers as shared among all phenotypes. We then branched the networks for phenotypes that are deemed dissimilar. Our basic rationale for such a design is to use 1) common shared layers to learn the pan-representations across multiple traits, 2) layers shared by branch to capture common characteristics among traits that are similar, and 3) task-specific layers to capture the uniqueness of each trait. This network architecture enables efficient information transfer among similar phenotypes while avoiding negative impacts on dissimilar ones.

## 3 Simulation studies

We conducted extensive simulations to assess the performance of our method. As outlined in the method, we pre-set the number of branches to be 2, 3, and 4, and then found the trait partition accordingly. We compared our model with a typical hard parameter sharing model where all parameters except the last are shared (denoted as HPS), a single-task model with each trait modeled independently (denoted as STL), and Multi-lasso that is a classic method for modeling multiple traits (Cheng et al., 2019). As we aim to improve prediction for multiple AD-related traits (i.e., FDG, AV45, FAQ, CDRSB, ADAS13, MMSE, and MoCA), we make our simulation settings like the ADNI dataset. Specifically, we directly extracted genomic data from ADNI and simulated seven phenotypes based on causal variants, which are harbored on six randomly selected genes and comprised of 10% of the total variants. We randomly split the samples into training, validation, and testing sets with a ratio of 8:1:1. For all models, we set 2 hidden layers with 128 and 32 hidden nodes, respectively (Supplementary Figure S1).

Since all simulated phenotypes are continuous, we used mean squared error (MSE) as the loss function during the model training process for all deep learning models. We evaluated the prediction performance based on the testing set and reported the average prediction Pearson correlations and root of MSE (RMSE) based on 100 Monte Carlo simulations under each setting. Pearson correlation captures the linear consistency between predicted and true values and is widely used in continuous phenotype prediction. RMSE complements this by quantifying the average prediction error in the same unit as the original data, providing a comprehensive assessment of model performance.

### 3.1 Scenario 1: the impact of different numbers of underlying groups among traits

In this scenario, we evaluated the impact of different numbers of underlying groups among the traits. We started with the case where all traits shared the same genetic causes (i.e., the underlying group is 1) and gradually split the traits into different groups until no traits shared any genetic causes (i.e., the underlying number of groups is 7).

Let $G_{j}$ denote the set of traits in the j -th group and $X_{G_{j}}$ is a $n\times p_{G_{j}}$ matrix of the corresponding causal variants for the j -th group, where n is the sample size. Note that in this set of simulations, there is no overlap between causal variants among different groups. When the number of underlying groups among the seven traits is one (i.e., $\left.G_{1}=\left\{1,2,3,4,5,6,7\right\}\right)$ , all traits share identical causes and are simulated as $y_{i}=X_{G_{1}}\beta +\epsilon_{i}$ for $\forall \;i\in G_{1}$ , where $\beta ∼N\left(0,I_{{p_{G}}_{1}}\sigma_{\beta }^{2}\right)$ and $\epsilon_{i}∼N\left(0,{I_{n}\sigma }_{\epsilon }^{2}\right)$ . When the number of underlying groups is seven, each trait has its own causes (i.e., $\left.G_{i}=\left\{i\right\}\right)$ and is simulated using $y_{i}=X_{G_{i}}\beta_{i}+\epsilon_{i}$ for $\forall \;i\in G_{i}$ with $\beta_{i}∼N\left(0,I_{{p_{G}}_{i}}\sigma_{\beta }^{2}\right).$ When the number of underlying groups is between two and six (i.e., some traits share identical causes), we simulated each trait as $y_{i}=X_{G_{j}}\beta_{G_{j}}+\epsilon_{i}$ for $\forall i\in G_{j}$ with $\beta_{G_{j}}∼N\left(0,I_{p_{G_{j}}}\sigma_{\beta }^{2}\right)$ . We split the seven traits given the specific number of groups and the details of grouping are summarized in Supplementary Table S1.

We varied effect sizes by ranging $\sigma_{\beta }/\sigma_{\epsilon }$ from 1:3 to 1:6. Table 1 presents the average Pearson correlations across the seven simulated traits, while the specific values for each trait are provided in Supplementary Tables S2–S5. When all traits have the same underlying causal factors, our model, regardless of the number of pre-specified number of branches, has similar performance to HPS, and it has much better performance than STL and Multi-Lasso model. For example, when $\sigma_{\beta }/\sigma_{\epsilon }$ = 1:5, the average Pearson correlations for Multi-Lasso, HPS, and STL are 0.266, 0.356, and 0.259, respectively. For our proposed method, they are 0.366, 0.368, and 0.355 when the respective pre-specified numbers of branches are two, three and four. Table 2 presents the average RMSE values for the seven simulated traits, with trait-specific results shown in Supplementary Tables S6–S9. These results indicate that our model also performs well in terms of RMSE. For example, under the $\sigma_{\beta }/\sigma_{\epsilon }$ = 1:5 setting, the RMSE of Multi-Lasso reaches 1.192, which is substantially higher than those of other methods; HPS and STL yield RMSEs of 0.918 and 0.976, respectively. In contrast, our model yields lower RMSEs of 0.914, 0.912, and 0.914 for branch numbers of two, three, and four, respectively, demonstrating lower prediction errors and confirming the robustness of our model in controlling estimation error.

**TABLE 1:** Average Pearson correlations for seven traits under different numbers of underlying groups among traits.

Sharing situations	Multi-lasso	HPS^a^	2 Groups^b^	3 Groups^c^	4 Groups^d^	STL^e^
$\sigma_{\beta }/\sigma_{\epsilon }$ = 1:3						
Complete Sharing	0.463	0.531	0.543	0.538	0.539	0.433
Two-group Sharing	0.425	0.478	0.495	0.491	0.494	0.377
Three-group Sharing	0.404	0.420	0.448	0.451	0.447	0.416
Four-group Sharing	0.402	0.402	0.417	0.417	0.422	0.398
No Sharing	0.393	0.322	0.332	0.331	0.328	0.395
$\sigma_{\beta }/\sigma_{\epsilon }$ = 1:4						
Complete Sharing	0.349	0.438	0.445	0.443	0.444	0.343
Two-group Sharing	0.313	0.377	0.394	0.394	0.392	0.319
Three-group Sharing	0.291	0.320	0.338	0.340	0.337	0.290
Four-group Sharing	0.293	0.294	0.310	0.312	0.313	0.300
No Sharing	0.289	0.220	0.234	0.235	0.226	0.291
$\sigma_{\beta }/\sigma_{\epsilon }$ = 1:5						
Complete Sharing	0.266	0.356	0.366	0.368	0.355	0.259
Two-group Sharing	0.239	0.274	0.305	0.301	0.304	0.254
Three-group Sharing	0.215	0.218	0.251	0.262	0.253	0.229
Four-group Sharing	0.220	0.199	0.228	0.237	0.235	0.230
No Sharing	0.218	0.140	0.147	0.138	0.155	0.219
$\sigma_{\beta }/\sigma_{\epsilon }$ = 1:6						
Complete Sharing	0.207	0.290	0.295	0.295	0.308	0.210
Two-group Sharing	0.183	0.218	0.239	0.242	0.235	0.186
Three-group Sharing	0.163	0.153	0.192	0.198	0.190	0.174
Four-group Sharing	0.169	0.129	0.159	0.172	0.174	0.173
No Sharing	0.171	0.078	0.098	0.088	0.096	0.169

^a^ Hard parameter sharing across all layers except the last one.

^b^ The pre-specified number of branches for the auto-branch method is two.

^c^ The pre-specified number of branches for the auto-branch method is three.

^d^ The pre-specified number of branches for the auto-branch method is four.

^e^ Each trait is modeled independently without accounting for trait correlations.

**TABLE 2:** Average RMSEs for seven traits under different numbers of underlying groups among traits.

Sharing situations	Multi-lasso	HPS^a^	2 Groups^b^	3 Groups^c^	4 Groups^d^	STL^e^
$\sigma_{\beta }/\sigma_{\epsilon }$ = 1:3						
Complete Sharing	1.001	0.832	0.829	0.829	0.831	0.893
Two-group Sharing	1.036	0.857	0.851	0.853	0.850	0.910
Three-group Sharing	1.054	0.890	0.878	0.880	0.879	0.908
Four-group Sharing	1.056	0.892	0.886	0.885	0.887	0.913
No Sharing	1.061	0.941	0.932	0.936	0.934	0.917
$\sigma_{\beta }/\sigma_{\epsilon }$ = 1:4						
Complete Sharing	1.115	0.882	0.879	0.879	0.876	0.937
Two-group Sharing	1.145	0.908	0.900	0.902	0.900	0.947
Three-group Sharing	1.162	0.936	0.930	0.927	0.927	0.960
Four-group Sharing	1.161	0.935	0.930	0.931	0.929	0.963
No Sharing	1.163	0.979	0.973	0.973	0.969	0.958
$\sigma_{\beta }/\sigma_{\epsilon }$ = 1:5						
Complete Sharing	1.192	0.918	0.914	0.912	0.914	0.976
Two-group Sharing	1.215	0.939	0.933	0.935	0.933	0.986
Three-group Sharing	1.232	0.967	0.958	0.958	0.961	0.974
Four-group Sharing	1.230	0.963	0.957	0.957	0.958	0.990
No Sharing	1.229	0.995	0.992	0.995	0.992	0.985
$\sigma_{\beta }/\sigma_{\epsilon }$ = 1:6						
Complete Sharing	1.246	0.942	0.936	0.937	0.939	0.993
Two-group Sharing	1.266	0.961	0.958	0.957	0.957	0.998
Three-group Sharing	1.278	0.983	0.979	0.976	0.977	1.000
Four-group Sharing	1.276	0.975	0.973	0.974	0.974	0.997
No Sharing	1.274	1.001	1.000	1.001	1.001	1.004

^a^ Hard parameter sharing across all layers except the last one.

^b^ The pre-specified number of branches for the auto-branch method is two.

^c^ The pre-specified number of branches for the auto-branch method is three.

^d^ The pre-specified number of branches for the auto-branch method is four.

^e^ Each trait is modeled independently without accounting for trait correlations.

When neither all traits share identical causes nor are completely independent of one another, our branching method tends to perform better than Multi-Lasso, HPS, and STL models, regardless of the pre-specified number of branches. For instance, when $\sigma_{\beta }/\sigma_{\epsilon }$ = 1:5 and the number of underlying groups of traits is two, the average Pearson correlations for Multi-Lasso, HPS, and STL models are 0.239, 0.274, and 0.254, respectively. For our auto-branch model, they are 0.305, 0.301 and 0.304 when the pre-specified branches are two, three and four, respectively. In terms of RMSE, our method also demonstrates superior performance. The average RMSEs for our model are 0.933, 0.935, and 0.933 for two, three, and four branches, respectively, all of which are lower than those of Multi-Lasso (1.215), HPS (0.939), and STL (0.986). This demonstrates that moderate grouping can effectively leverage shared information between traits, thereby enhancing prediction performance. It is worth noting that although our proposed method performs the best when the pre-specified number of branches matches the underlying disease model, the difference resulting from this is relatively small, indicating the robustness of our method in most practical applications.

Although it is highly unlikely that disease-relevant correlated traits have no shared genetic causes, we still assessed this situation for completeness. As expected, when each trait has its own causes, jointly training cannot benefit model performance. The single-task STL has the best performance, followed by the Multi-Lasso model. Our method performs similarly to that of HPS, as our model assumes that traits share some common causes and sets the pre-specified number of branches smaller than the true underlying groups. Although we can specify the number of branches to be exactly the same as the number of traits, we considered this unnecessary in practice, as only traits that are expected to share some underlying causes should be dealt with using the MTL approach.

In summary, models assuming either identical causes for all traits or complete independence perform poorly when these assumptions are violated. HPS performs well when all traits share the same causes, but its performance drops sharply as the degrees of sharedness among traits decreases. For STL that assumes traits are independent of each other, it has outperformed the models where grouping is not needed, but it suffers greatly when indeed some phenotypes share identical causes. Multi-Lasso allows for independent feature selection for each trait, which enables competitive performance when traits are largely unrelated. However, in scenarios where traits share underlying causal factors, the model may not fully exploit such shared structures due to the lack of an explicit trait grouping mechanism. For our proposed method, it can have robust and better performance, provided the traits are not completely independent of each other. We consider this property important for the prediction of multiple disease-related traits. For example, within the AD prediction domain, it is highly unlikely that AD-related traits are completely independent and thus STL that fails to account for their relatedness can have sub-optimal performance. Similarly, as the amount of sharedness among these AD-related traits is unknown in advance, HPS is also unlikely to achieve the best performance. A method that can flexibly account for different degrees of sharedness can be of great use for these studies.

### 3.2 Scenario 2: the impact of the relative contributions of shared causal variants

In practice, correlated traits can not only have shared causes, but also their own unique risk factors. In this set of simulations, we evaluated the relative contributions of shared causal factors on the model performance. We used $X_{i}$ and $X_{s}$ to respectively denote $n\times p_{i}$ and $n\times p_{s}$ matrices, representing causal factors specific to trait *i* and shared across traits. Similar to scenario 1, we considered seven traits, and simulated the outcome under the additive model as $y_{i}=X_{i}\beta_{i}+X_{s}\beta_{s}+\epsilon_{i}$ , where $\beta_{i}∼N\left(0,{I_{p_{i}}\sigma }_{\beta }^{2}\right)$ , $\beta_{s}∼N\left(0,{I_{p_{s}}\sigma }_{\beta_{s}}^{2}\right)$ , and $\epsilon_{i}∼N\left(0,{I_{n}\sigma }_{\epsilon }^{2}\right)$ . We gradually varied the relative importance of the unique risk factors to the shared ones and set $\sigma_{\beta }^{2}/\sigma_{\beta_{s}}^{2}$ at 1:9, 3:7, 5:5, 7:3, and 9:1, indicating a gradual increase in the influence of the shared causal variants. We also varied the effect sizes of all causal variants, where the signal-to-noise ratio (i.e., $\left.\left(\sigma_{\beta }^{2}+\sigma_{\beta_{s}}^{2}\right)/\sigma_{\epsilon }^{2}\right)$ is set at 1:9, 1:16, 1:25, and 1:36.

Table 3 presents the average Pearson correlations across the seven simulated traits as the amount of shared causal factors varies and the specific values for each trait are provided in Supplementary Tables S10–S13. Table 4 shows the corresponding average RMSE values, with detailed results listed in Supplementary Tables S14–S17. When shared causal variants significantly contribute to the outcomes, our method substantially enhances the prediction. For example, when $\sigma_{\beta }^{2}/\sigma_{\beta_{s}}^{2}$ = 1:9 and signal-to-noise ratio is 1:25, the average Pearson correlation achieved through the auto-branch method are 0.285, 0.293, and 0.286 when the pre-specified number of branches are 2, 3, and 4, respectively. The corresponding RMSEs are all 0.933. In contrast, Multi-Lasso, STL, and HPS obtain lower correlations (0.215, 0.232, and 0.271) and higher RMSEs (1.239, 0.967, and 0.935). When $\sigma_{\beta }^{2}/\sigma_{\beta_{s}}^{2}$ = 3:7, a similar trend remains. This indicates that, when shared causal factors explain most of the variabilities in the traits, allowing for grouping to enable information sharing can enhance model performance. HPS generally performs worse than our proposed method, indicating allowing uniqueness for traits when they are not caused by the same factors is important for the improved prediction.

**TABLE 3:** Average Pearson correlations for seven traits as the relative contributions between unique causal factors and shared causal factors increases.

$\sigma_{\beta }^{2}/\sigma_{\beta_{s}}^{2}$	Multi-lasso	HPS^a^	2 Groups^b^	3 Groups^c^	4 Groups^d^	STL^e^
$\left(\sigma_{\beta }^{2}+\sigma_{\beta_{s}}^{2}\right)/\sigma_{\epsilon }^{2}$ = 1:9						
1:9	0.444	0.495	0.501	0.502	0.504	0.416
3:7	0.440	0.428	0.444	0.450	0.444	0.411
5:5	0.429	0.404	0.418	0.420	0.414	0.400
7:3	0.350	0.390	0.402	0.403	0.407	0.395
9:1	0.357	0.329	0.349	0.353	0.348	0.369
$\left(\sigma_{\beta }^{2}+\sigma_{\beta_{s}}^{2}\right)/\sigma_{\epsilon }^{2}$ = 1:16						
1:9	0.324	0.397	0.405	0.418	0.416	0.326
3:7	0.306	0.335	0.345	0.345	0.343	0.297
5:5	0.314	0.294	0.319	0.314	0.313	0.295
7:3	0.299	0.245	0.266	0.264	0.271	0.280
9:1	0.299	0.234	0.247	0.250	0.245	0.273
$\left(\sigma_{\beta }^{2}+\sigma_{\beta_{s}}^{2}\right)/\sigma_{\epsilon }^{2}$ = 1:25						
1:9	0.215	0.271	0.285	0.293	0.286	0.232
3:7	0.228	0.268	0.284	0.281	0.282	0.222
5:5	0.217	0.223	0.243	0.238	0.234	0.224
7:3	0.214	0.151	0.177	0.192	0.188	0.203
9:1	0.216	0.134	0.155	0.161	0.158	0.215
$\left(\sigma_{\beta }^{2}+\sigma_{\beta_{s}}^{2}\right)/\sigma_{\epsilon }^{2}$ = 1:36						
1:9	0.201	0.259	0.269	0.273	0.269	0.200
3:7	0.187	0.212	0.223	0.227	0.225	0.177
5:5	0.176	0.152	0.177	0.179	0.179	0.170
7:3	0.187	0.131	0.152	0.152	0.154	0.177
9:1	0.168	0.086	0.107	0.110	0.111	0.155

^a^ Hard parameter sharing across all layers except the last one.

^b^ The pre-specified number of branches for the auto-branch method is two.

^c^ The pre-specified number of branches for the auto-branch method is three.

^d^ The pre-specified number of branches for auto-branch method is four.

^e^ Each trait is modeled independently without accounting for trait correlations.

**TABLE 4:** Average RMSEs for seven traits as the relative contributions between unique causal factors and shared causal factors increases.

$\sigma_{\beta }^{2}/\sigma_{\beta_{s}}^{2}$	Multi-lasso	HPS^a^	2 Groups^b^	3 Groups^c^	4 Groups^d^	STL^e^
$\left(\sigma_{\beta }^{2}+\sigma_{\beta_{s}}^{2}\right)/\sigma_{\epsilon }^{2}$ = 1:9						
1:9	1.019	0.852	0.851	0.851	0.849	0.897
3:7	1.028	0.886	0.882	0.882	0.882	0.899
5:5	1.040	0.905	0.900	0.903	0.899	0.906
7:3	1.038	0.918	0.912	0.912	0.913	0.904
9:1	1.054	0.933	0.926	0.928	0.926	0.921
$\left(\sigma_{\beta }^{2}+\sigma_{\beta_{s}}^{2}\right)/\sigma_{\epsilon }^{2}$ = 1:16						
1:9	1.141	0.899	0.899	0.894	0.898	0.937
3:7	1.149	0.930	0.925	0.923	0.924	0.944
5:5	1.146	0.950	0.944	0.944	0.948	0.951
7:3	1.155	0.965	0.961	0.961	0.960	0.945
9:1	1.156	0.971	0.966	0.961	0.967	0.960
$\left(\sigma_{\beta }^{2}+\sigma_{\beta_{s}}^{2}\right)/\sigma_{\epsilon }^{2}$ = 1:25						
1:9	1.239	0.935	0.933	0.933	0.933	0.967
3:7	1.222	0.953	0.948	0.948	0.951	0.971
5:5	1.216	0.961	0.959	0.959	0.959	0.976
7:3	1.230	0.978	0.975	0.975	0.977	0.978
9:1	1.231	0.990	0.989	0.988	0.992	0.987
$\left(\sigma_{\beta }^{2}+\sigma_{\beta_{s}}^{2}\right)/\sigma_{\epsilon }^{2}$ = 1:36						
1:9	1.254	0.952	0.951	0.948	0.947	0.991
3:7	1.263	0.960	0.957	0.957	0.957	0.995
5:5	1.271	0.975	0.975	0.973	0.973	0.999
7:3	1.257	0.982	0.979	0.981	0.978	0.994
9:1	1.272	0.995	0.994	0.993	0.994	1.009

^a^ Hard parameter sharing across all layers except the last one.

^b^ The pre-specified number of branches for the auto-branch method is two.

^c^ The pre-specified number of branches for the auto-branch method is three.

^d^ The pre-specified number of branches for auto-branch method is four.

^e^ Each trait is modeled independently without accounting for trait correlations.

When shared and non-shared causal factors contribute equally, a well-considered branching strategy can still help the model better capture the distinctions between traits, thereby improving predictions as compared to both STL and HPS. In this scenario, although Multi-Lasso shows comparable performance to our method in terms of Pearson correlation, it exhibits higher RMSE values, indicating its relative limitation in error control. However, when trait-specific factors make major contributions to the outcome variability, while the prediction performance after branching increases as compared to HPS, it does not surpass those in the STL or Multi-Lasso models. This suggests that, in such cases, the independence between traits renders group training less effective and negative information transfer may occur. In contrast, methods like STL or joint models with independent feature selection pathways, such as Multi-Lasso, are more appropriate in scenarios with minimal shared information. Therefore, when traits are expected to be largely independent, STL or similar strategies should be considered as the first choice and multi-task learning is not expected to benefit model performance.

Overall, joint modeling benefits more when the shared factors substantially contributed to correlated traits, whereas separate modeling and Multi-Lasso would be preferred if trait-specific factors explain most of the variability. In practical MTL applications, traits often share a moderate amount of common causes. Under these circumstances, our method has a notable advantage over HPS and STL by effectively utilizing shared information while preserving trait-specific focus, and it also outperforms Multi-Lasso, which fails to fully exploit the shared structure, enhancing its practical utility.

## 4 The prediction analyses for multiple AD-related traits

We are interested in predicting multiple AD-related traits, including cognitive scores (i.e., MMSE, MoCA, ADAS13), functional assessments (i.e., FAQ, CDRSB), and neuroimaging findings (i.e., AV45 and FDG), using genetic data obtained from ADNI. ADNI is a comprehensive longitudinal study aimed at identifying biomarkers associated with AD and enhancing its clinical diagnosis and early intervention. As all seven phenotypes are quantitatively measured continuous traits, they were modeled as regression tasks in our study.

Data were downloaded from the ADNI website (https://www.adni.loni.usc.edu/). We excluded individuals without genomic data or have missing phenotypes. Only autosome SNPs were considered in our analysis. We adopted a candidate gene approach, where 57 AD susceptibility genes identified based on existing literature were included (Supplementary Table S18). For quality control, we excluded SNPs if they met any of the subsequent criteria: 1) missing rate >1%; 2) minor allele frequency (MAF) < 5%; 3) Hardy-Weinberg equilibrium test with p -value <1 × 10^−6^; 4) linkage disequilibrium (LD) analysis with an LD threshold of R^2^ > 0.9. Additionally, individuals with missing rate of SNPs larger than 1% were excluded. All selected SNPs were directly genotyped in the ADNI dataset. For individuals who passed the quality control, we utilized the plink2R package to impute the missing for the included SNPs. A total of 463 participants and 3,797 SNPs harbored on the 57 AD susceptibility genes were included in the final analyses. The distributions of seven phenotypes are shown in Supplementary Figure S2.

We randomly split the data into an 8:1:1 ratio for training, validation and testing to mitigate overfitting, and repeated the random sampling 100 times for robustness. Given that all seven traits are AD-related and some even focus on similar aspect of AD (e.g., cognitive changes), it is unlikely they are fully independent. Likewise, while these traits provide complementary insights into AD, they are unlikely to share identical genetic causes. Therefore, for our method, we set the pre-specified number of branches to be 2, 3, and 4, excluding cases where all traits are independent or share identical causes. For comparison, we included the Multi-Lasso model, the hard sharing model HPS, where all layers are shared except for the last, and the STL model, where each trait is trained separately. We kept the network architecture consistent with that used in the simulations. We reported Pearson correlation and RMSE for all methods and further used Wilcoxon signed-rank test to compare our method to the others.

**FIGURE 2:**
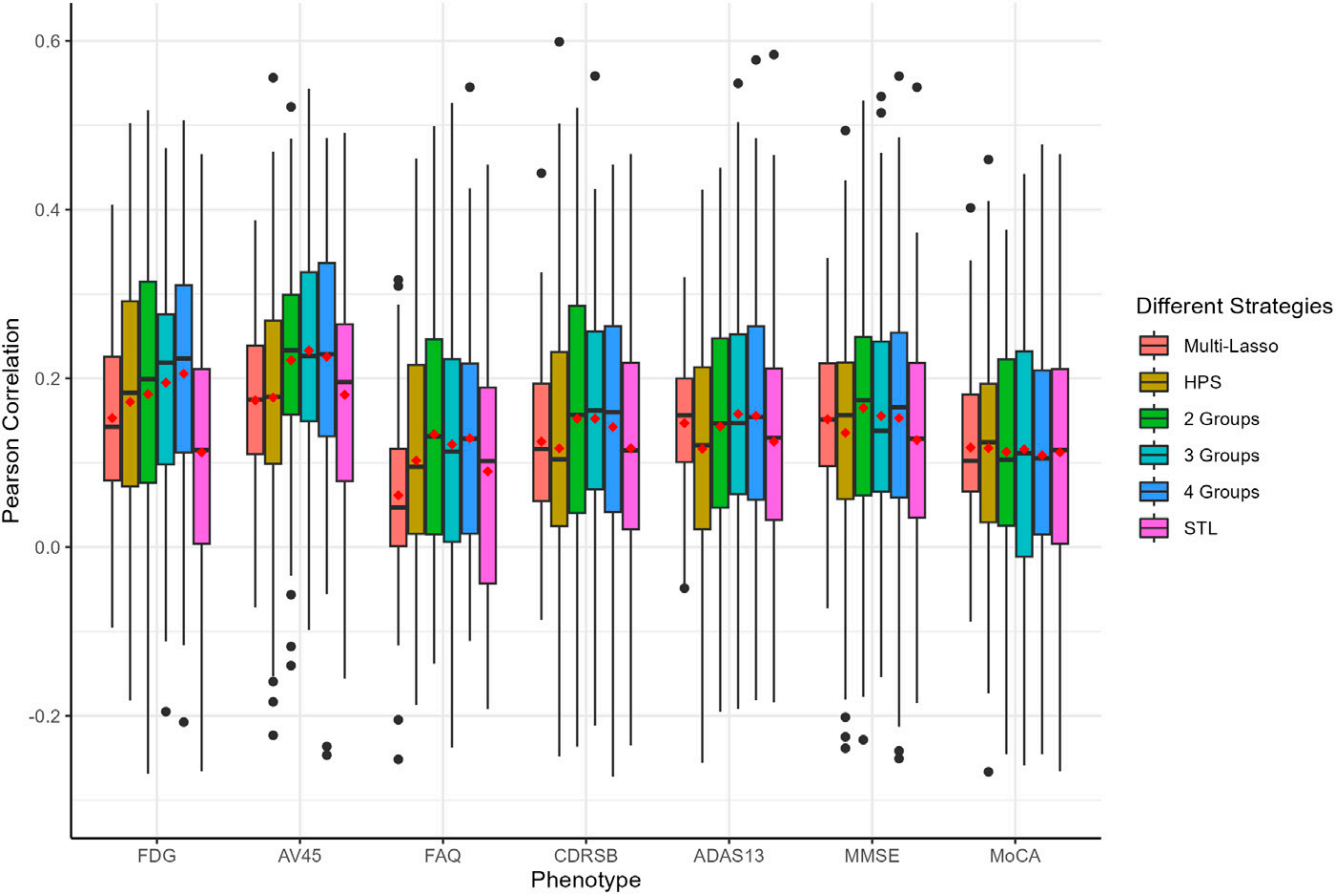
Comparison of prediction performance of multiple AD-related traits using Pearson correlation. The red dots in the figure represent the average Pearson correlations. HPS: Hard parameter sharing across all layers except the last one. 2 Groups: The pre-specified number of branches for auto-branch method is two. 3 Groups: The pre-specified number of branches for auto-branch method is three. 4 Groups: The pre-specified number of branches for auto-branch method is four. STL: Each trait is modeled independently without accounting for trait correlations. Phenotypes include fluorodeoxyglucose (FDG) and florbetapir (AV45) PET imaging, Functional Activities Questionnaire (FAQ), Clinical Dementia Rating-Sum of Boxes (CDRSB) Alzheimer’s Disease Assessment Scale-Cognitive Subscale 13 (ADAS13), Mini-Mental State Examination (MMSE), and Montreal Cognitive Assessment (MoCA).

**FIGURE 3:**
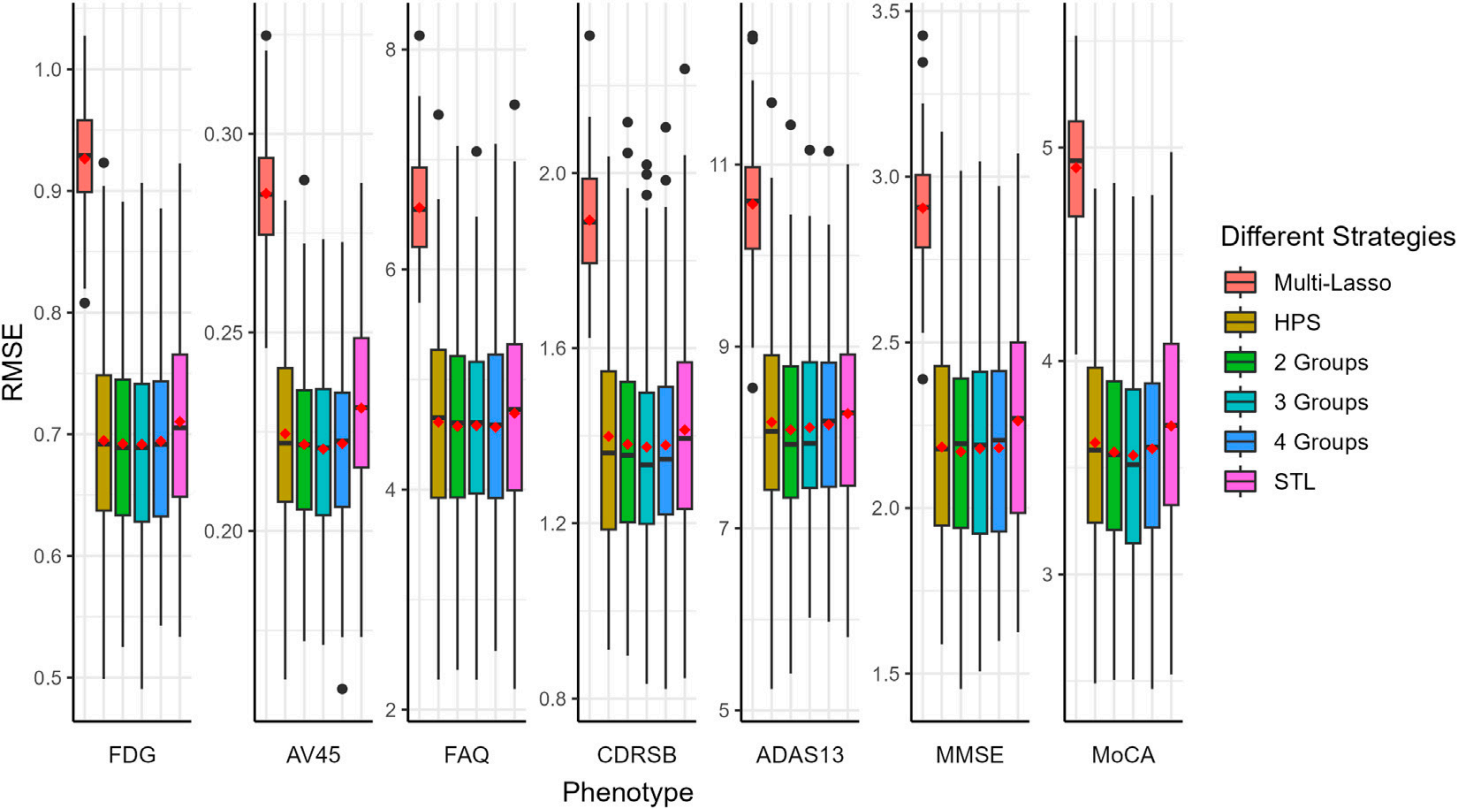
Comparison of prediction performance of multiple AD-related traits using RMSE. The red dots in the figure represent the average RMSEs. HPS: Hard parameter sharing across all layers except the last one. 2 Groups: The pre-specified number of branches for auto-branch method is two. 3 Groups: The pre-specified number of branches for auto-branch method is three. 4 Groups: The pre-specified number of branches for auto-branch method is four. STL: Each trait is modeled independently without accounting for trait correlations. Phenotypes include fluorodeoxyglucose (FDG) and florbetapir (AV45) PET imaging, Functional Activities Questionnaire (FAQ), Clinical Dementia Rating-Sum of Boxes (CDRSB) Alzheimer’s Disease Assessment Scale-Cognitive Subscale 13 (ADAS13), Mini-Mental State Examination (MMSE), and Montreal Cognitive Assessment (MoCA).

The prediction performance, measured by Pearson correlation and RMSE, is illustrated in Figures 2, 3, respectively. Our auto-branch method performs the best when the pre-specified number of branches is set to three, though the performance differences across various branch numbers are minimal. This method demonstrates superior prediction across multiple traits, not only in terms of higher average Pearson correlations but also by maintaining lower average RMSEs. Among all compared methods, Multi-Lasso demonstrates weaker performance in several key traits (Table 5). Specifically, when the number of branches is set to three, the average Pearson correlations for FDG, AV45, FAQ, CDRSB, and ADAS13 show relative improvements of 29.45%, 33.96%, 98.60%, 17.48%, and 7.51% over Multi-Lasso, with corresponding absolute increases of 0.045, 0.059, 0.061, 0.022, and 0.011, respectively. In addition, Multi-Lasso yields consistently higher RMSEs, particularly for FAQ and ADAS13, reflecting its limited capacity to model complex inter-trait relationships. The Wilcoxon signed-rank test indicated that the increase is statistically significant for FDG, AV45, and FAQ (Supplementary Table S19).

**TABLE 5:** Predictive performance (average Pearson correlations and RMSEs) for seven Alzheimer’s-related phenotypes on real-world data.

Different strategies	Seven Alzheimer’s related phenotypes^a^						
	FDG	AV45	FAQ	CDRSB	ADAS13	MMSE	MOCA
Pearson correlation							
Multi-Lasso	0.153	0.174	0.061	0.125	0.147	0.152	0.118
HPS^b^	0.167	0.177	0.092	0.111	0.116	0.121	0.117
2 Groups^c^	0.181	0.226	0.134	0.152	0.143	0.165	0.113
3 Groups^d^	0.198	0.233	0.122	0.147	0.158	0.151	0.116
4 Groups^e^	0.200	0.226	0.134	0.142	0.156	0.153	0.108
STL^f^	0.112	0.181	0.090	0.122	0.130	0.127	0.112
RMSE							
Multi-Lasso	0.926	0.285	6.563	1.892	10.563	2.906	4.906
HPS	0.694	0.225	4.613	1.399	8.169	2.184	3.617
2 Groups	0.692	0.222	4.575	1.381	8.084	2.170	3.573
3 Groups	0.692	0.221	4.581	1.374	8.109	2.179	3.558
4 Groups	0.694	0.222	4.566	1.378	8.140	2.181	3.589
STL	0.710	0.231	4.693	1.414	8.262	2.263	3.695

^a^ Phenotypes include fluorodeoxyglucose (FDG) and florbetapir (AV45) PET, imaging, Functional Activities Questionnaire (FAQ), Clinical Dementia Rating-Sum of Boxes (CDRSB) Alzheimer’s Disease Assessment Scale-Cognitive Subscale 13 (ADAS13), Mini-Mental State Examination (MMSE), and Montreal Cognitive Assessment (MoCA).

^b^ Hard parameter sharing across all layers except the last one.

^c^ The pre-specified number of branches for the auto-branch method is two.

^d^ The pre-specified number of branches for the auto-branch method is three.

^e^ The pre-specified number of branches for auto-branch method is four.

^f^ Each trait is modeled independently without accounting for trait correlations.

Regardless of branch number, our method consistently performs similarly to or better than HPS (i.e., similar or higher Pearson correlations, and similar or lower RMSE). Specifically, the improvements in average Pearson correlations for FDG, AV45, FAQ, CDRSB, ADAS13, and MMSE with a three-branch setup are 18.56%, 31.64%, 32.61%, 32.43%, 36.21%, and 24.79%, with corresponding absolute increases of 0.031, 0.056, 0.030, 0.036, 0.042, and 0.030, respectively. The Wilcoxon signed-rank test indicated that the increase is statistically significant for AV45, CDRSB, and ADAS13 (Supplementary Table S19).

Compared to the STL models, our method consistently performs similarly to or better than STL (i.e., similar or higher Pearson correlations and consistently lower RMSE). The improvements in average Pearson correlations for FDG, AV45, FAQ, CDRSB, ADAS13, MMSE, and MoCA are 76.18%, 29.07%, 35.78%, 20.36%, 21.62%, 19.03%, and 3.22%, respectively, with corresponding absolute increases of 0.086, 0.052, 0.032, 0.025, 0.028, 0.024, and 0.004. The Wilcoxon signed-rank test indicated a statistically significant increase for FDG, AV45, FAQ, and MMSE (Supplementary Table S19).

Our analyses suggest that the seven AD-related traits neither share identical genetic causes nor are completely independent. PET-imaging traits FDG and AV45 benefit substantially from joint modeling, indicating cognitive and function tests provide auxiliary information. Information transfer between PET-imaging outcomes and cognitive as well as function tests help learn a better representation, leading to the improvement of prediction. In summary, our method improves prediction performance for most of the traits regardless of the pre-specified number of branches, highlighting its robustness and potential for broad practical applications, especially for phenotypes where identifying latent patterns is essential.

**FIGURE 4:**
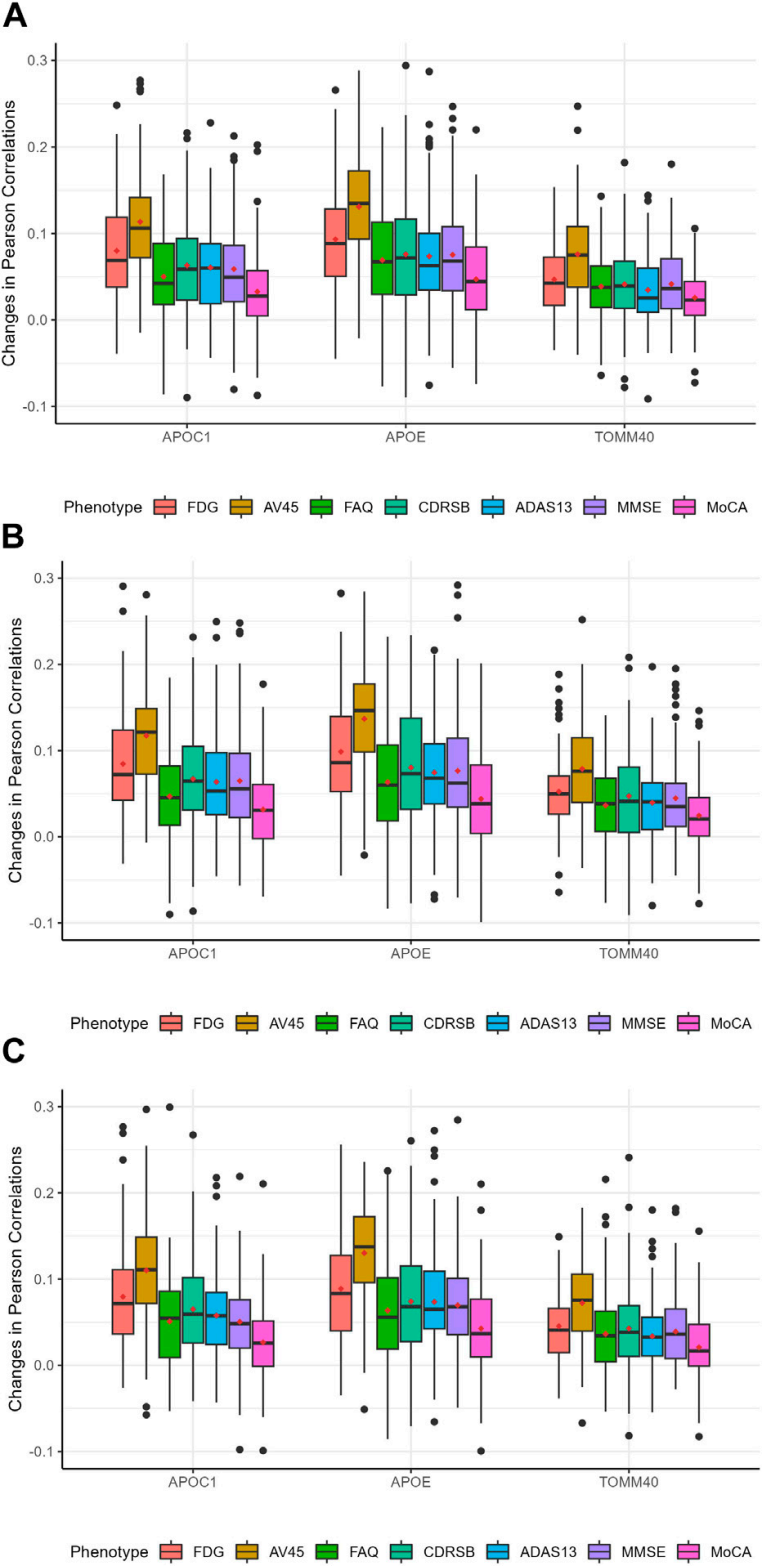
Distribution of predictive feature importance scores for the top significant genes. The red dots in the figure represent the average Pearson correlations. **(A)**: The pre-specified number of branches for the auto-branch method is two. **(B)**: The pre-specified number of branches for the auto-branch method is three. **(C)**: The pre-specified number of branches for the auto-branch method is four. Phenotypes include fluorodeoxyglucose (FDG) and florbetapir (AV45) PET imaging, Functional Activities Questionnaire (FAQ), Clinical Dementia Rating-Sum of Boxes (CDRSB) Alzheimer’s Disease Assessment Scale-Cognitive Subscale 13 (ADAS13), Mini-Mental State Examination (MMSE), and Montreal Cognitive Assessment (MoCA).

To further investigate our model, we calculated the predictive feature importance score for each gene using a permutation-based approach proposed by Liu et al. (2022). The basic rationale is that if a gene is predictive, then the model accuracies with and without it would differ significantly. Following the procedure proposed by Liu et al. (2022), we assessed the importance of each gene by quantifying the difference in accuracies while accounting for variability. Specifically, we first calculated the Pearson correlation between predicted and observed outcomes using the original data with the already trained auto-branch model. We then recalculated the Pearson correlations after randomly shuffling the SNPs located within the gene of interest, while preserving the genetic structure (e.g., linkage disequilibrium), to generate a null distribution. To empirically estimate the variance, we repeated the permutation process 100 times. The standardized difference in Pearson correlations between the original and shuffled data (also called the predictive feature importance score by Liu et al.) reflects the gene’s contribution to predictive performance, with a larger difference indicating greater predictive importance. We then calculated the predictive feature importance score, which follows an asymptotic normal distribution under the null as shown by Liu et al. (2022), to evaluate the predictive importance of each gene. Genes were ranked based on their predictive importance scores, and we focused on those with scores greater than 1.645, corresponding to a 5% significance threshold under the asymptotic normal distribution. It is important to note that we did not intend to perform hypothesis testing, rather we chose a cut-off value (i.e., 1.645) and focused on genes with predictive feature importance score larger than this cut-off. Therefore, no multiple testing correction was applied. Table 6 presents the genes identified as having a probability greater than 75% of being significantly predictive at the 5% level for at least one trait, and the details for each gene are provided in Supplementary Tables S20–S22, which correspond to models with 2, 3, and 4 pre-specified numbers of branches, respectively. *APOC1*, *APOE*, and *TOMM40* demonstrate stable and significant predictive power. Even though the most significant genes are similar among phenotypes, their predictive power varies considerably (Figure 4), which explains the superior performance of our branching method over HPS. In our model with three branches, *APOE* plays a crucial role in predicting FDG and AV45, with its removal leading to average Pearson correlations decreases of 0.10 and 0.14, respectively. For FAQ, CDRSB, ADAS13, MMSE and MoCA, the impact is small-to-moderate, with Pearson correlations reduced by 0.06, 0.08, 0.08, 0.08, and 0.04 respectively.

**TABLE 6:** Probability of the gene being significantly predictive at 5% level with probably for at least one trait greater than 75%.

Different strategies	Gene	Seven Alzheimer’s related phenotypes^a^						
		FDG	AV45	FAQ	CDRSB	ADAS13	MMSE	MoCA
2 Groups^b^	*APOC1*	0.92	0.98	0.87	0.87	0.85	0.85	0.74
	*APOE*	0.94	0.97	0.86	0.87	0.90	0.85	0.77
	*TOMM40*	0.85	0.93	0.79	0.82	0.77	0.83	0.73
3 Groups^c^	*APOC1*	0.93	0.98	0.77	0.86	0.88	0.84	0.68
	*APOE*	0.95	0.96	0.81	0.89	0.90	0.87	0.72
	*TOMM40*	0.91	0.93	0.77	0.78	0.76	0.80	0.71
4 Groups^d^	*APOC1*	0.89	0.96	0.75	0.87	0.87	0.84	0.70
	*APOE*	0.90	0.95	0.83	0.84	0.89	0.90	0.77
	*TOMM40*	0.86	0.93	0.73	0.78	0.78	0.78	0.70

^a^ Phenotypes include fluorodeoxyglucose (FDG) and florbetapir (AV45) PET, imaging, Functional Activities Questionnaire (FAQ), Clinical Dementia Rating-Sum of Boxes (CDRSB) Alzheimer’s Disease Assessment Scale-Cognitive Subscale 13 (ADAS13), Mini-Mental State Examination (MMSE), and Montreal Cognitive Assessment (MoCA).

^b^ The pre-specified number of branches for the auto-branch method is two.

^c^ The pre-specified number of branches for the auto-branch method is three.

^d^ The pre-specified number of branches for the auto-branch method is four.

## 5 Discussion

In this study, we proposed an efficient and robust auto-branch multi-task learning method for simultaneously predicting multiple correlated traits. Using total inter-task affinity, which quantifies the impact of gradient updates from one trait on the others, our method automatically determines the best partition of traits to enable efficient information transfer among similar traits, thereby enhancing prediction performance.

Through simulations, we found that our method has similar or better performance than that of the hard parameter sharing model where shared layers are pre-specified (Zhao et al., 2020). Our auto-branch model identifies the optimal phenotype partitioning that maximizes overall inter-trait affinity. Phenotypes grouped together share layers, while those in separate groups are assigned distinct branches. This data-driven approach to sharing layers enables efficient information transfer among inherently similar phenotypes and significantly reduces the risk of negative transfer. Therefore, our method can facilitate the capture of complex patterns in the data, which includes not only the shared representations but also the uniqueness of each phenotype. Our auto-branch method can also outperform the single-task models, when phenotypes shared moderate levels of common causes. This is mainly because single-task models fail to utilize information from auxiliary tasks and the efficient sample sizes are much less than those in the multi-task settings. Note that we designed our prediction model using a branch network architecture as opposed to training separate HPSs for each group of phenotypes. The rationale for such a design lies in the fact that most disease-related traits have shared genetic architecture (Badré and Pan, 2023), and by allowing all disease-related phenotypes to have some shared layers to enable the efficient modeling of these common patterns. In the unlikely event that the underlying genetic architectures differ significantly among traits in different groups, our proposed inter-trait affinity measure can still guide trait groupings. Separate HPS models can then be applied to each trait group for predictions (Supplementary Figure S3). This network structure facilitates capturing the unique characteristics of each group, making it particularly powerful for analyzing phenotypes that are largely distinct (Supplementary Tables S23, S24). Nevertheless, we recommend using branch network structure as illustrated in Figure 1 for genetic risk prediction of multiple correlated traits in most practical applications.

Our proposed method offers significant advantages in predicting seven AD-related phenotypes, including neuroimaging findings, cognitive scores, and functional assessments. For example, in the prediction of FDG, our method with 3 pre-determined branches increases the average Pearson correlations by 29.45%, 18.56% and 76.18% for Multi-Lasso, HPS, and STL, with corresponding absolute increase of 0.059, 0.031 and 0.086, respectively. Similarly, in the prediction of AV45, we have observed an increase of 33.96%, 31.64% and 29.07% for Multi-Lasso, HPS, and STL, with corresponding absolute increase of 0.061, 0.056 and 0.052, respectively. Notably, MoCA did not show significant improvement across the models, which may suggest weaker correlations with other traits or higher noise in its measurements that impacted model performance.

To verify the reliability and effectiveness of our proposed model, we conducted a comparative analysis with several related studies. For instance, Zhu et al. (2016) incorporated SNPs from the top 10 *APOE*-related genes into their MMSE prediction model and achieved a Pearson correlation of 0.150. In contrast, our method attained a higher correlation of 0.165 on the same task, demonstrating improved predictive performance. Hongmyeong-eup (2015) selected 39 SNPs, identified from approximately 1.5 million candidates as being closely associated with Alzheimer’s disease (AD) progression, and achieved a Pearson correlation of 0.400 in MMSE prediction. To ensure a fair comparison, we also constructed a model using SNPs from three well-established AD-associated genes—*APOC1*, *APOE*, and *TOMM40*—and obtained a correlation of 0.390, indicating that our approach performs comparably when using similarly strong genetic signals. In addition, Hao et al. (2016) reported Pearson correlation values ranging from 0.02 to 0.25 and 0.03–0.23 for traditional and improved methods on AV45 and FDG phenotypes, respectively. Our model also falls within these performance ranges, suggesting comparable accuracy in these tasks. In summary, our proposed method exhibited comparable predictive capabilities to existing state-of-the-art methods in multiple AD-related phenotypes, further demonstrating its potential in modeling Alzheimer’s disease progression.

These performance gains can be largely attributed to the underlying design of our method. Our method is constructed within a deep learning framework, effectively parsing complex trait relationships, particularly in the context of multi-gene co-regulation. Additionally, it can dynamically determine shared layers and thus is more powerful in managing intricate relationships among traits. Evidence suggests that these AD-related traits reflect different aspects of AD, and they are neither entirely correlated nor completely independent. For example, cognitive decline and neuroimaging abnormalities often exhibit strong genetic correlations (Zhang et al., 2021), but they do not share identical genetic causes. Therefore, methods like Multi-Lasso that rely on fixed feature selection across all tasks are unable to capture these nuanced relationships, as they do not allow for the dynamic identification of shared and unique factors between traits. Similarly, both STL and HPS models are unlikely to achieve the optimal performance, as STL fails to exploit inter-task correlations and HPS pre-specifies shared layers that lead to limited flexibility. Therefore, for the prediction of AD-related traits, our auto-branch multi-task learning method can leverage shared signals among genes or biological pathways to gain a more comprehensive understanding of these phenotypes while allowing each phenotype to have their unique characteristics, and thus offers greater flexibility as compared to Multi-Lasso, STL, and HPS.

We found that the *APOC1*, *APOE*, and *TOMM40* exhibit stable and significant predictive abilities for all seven AD-related phenotypes, but their predictive capabilities vary substantially across traits, indicating HPS that forces most of the model parameter the same is unlikely to work well. All highly predictive genes are significantly associated with AD. For example, APOE4*ε4 regulates neuronal metabolism and epigenetics, and is involved in the pathological processes of AD (Prasad and Rao, 2018). *APOC1* influences AD development through its role in cholesterol metabolism (Leduc et al., 2010), with the *APOC1 H2* allele potentially acting synergistically with *APOE* to increase the risk of cognitive decline (Zhou et al., 2014). *TOMM40* is strongly linked to *APOE* and contributes to the pathological changes in AD (Tasaki et al., 2019), including the formation of neurofibrillary tangles and neuritic plaques. *TOMM40* regulates oxidative stress and mitochondrial function, and is associated with late-onset AD (Roses, 2010). We noticed that the predictive ability of *APOE* for FDG and AV45 is especially significant, aligning with findings from previous research (Prasad and Rao, 2018). Future studies are needed to further decipher additional factors that contribute to the variability of each phenotype.

While this study offers valuable insights into the effectiveness of multi-task learning, several limitations remain. Due to the NP-hard nature of the problem, we pre-specified the optimal number of branches and then found the corresponding partitions. Future research can improve this by determining the optimal number of branches using a data-driven manner (e.g., cross-validation). Although our method outperforms baseline approaches, factors such as limited sample size, low-to-median heritability, and trait heterogeneity may still contribute to variability and should be further explored in future work. Additionally, the findings presented in this study are based on simulated data and ADNI dataset. Future work should validate these results across diverse datasets and applications.

In summary, we developed an efficient auto-branch multi-task learning framework for the prediction analyses of multiple correlated phenotypes. It can dynamically branch the network to allow for efficient information transfer and improve the overall prediction. Our method is available at https://github.com/jiaqi69/TAB.

## Data Availability

The original contributions presented in the study are included in the article/Supplementary Material, further inquiries can be directed to the corresponding authors.
