# Supplementary material for: Auto-branch multi-task learning for simultaneous prediction of multiple correlated traits associated with Alzheimer’s disease
Source: Front Genet. 2025 Jun 10;16:1538544. doi: 10.3389/fgene.2025.1538544 (PMC12185508; doi:10.3389/fgene.2025.1538544)
Supplement: Supplementary file 1 [file DataSheet1.pdf]

Supplementary Material

1 Supplementary Figures and Tables

1.1 Supplementary Figures

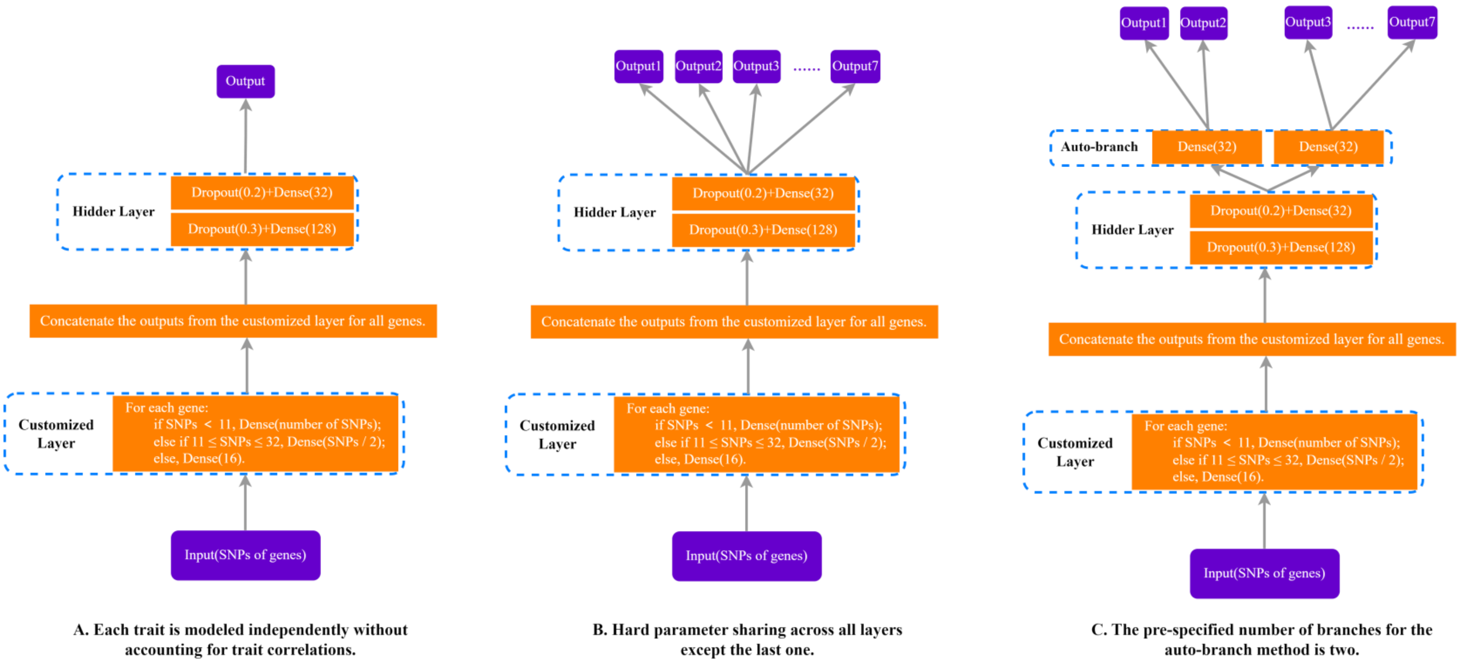

Supplementary Figure S1. Network architecture.

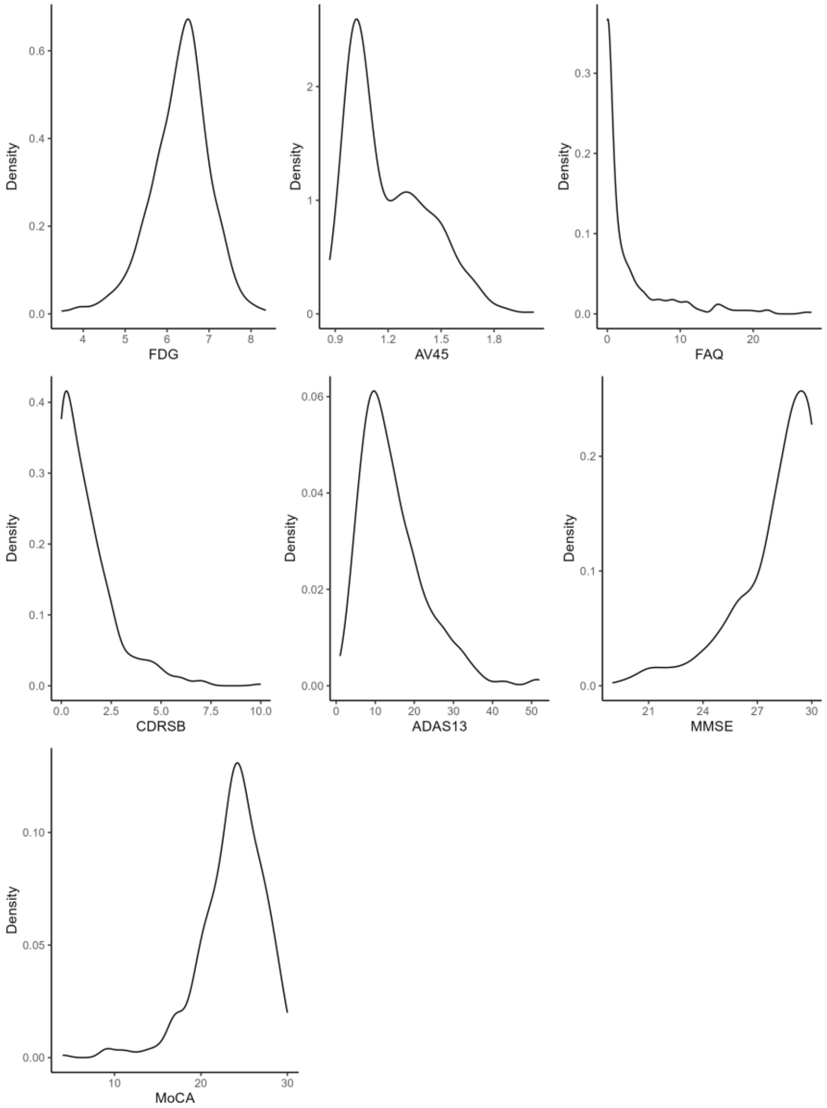

Supplementary Figure S2. Distributions for Alzheimer’s disease related phenotypes. Phenotypes include fluorodeoxyglucose (FDG) and florbetapir (AV45) PET imaging, Functional Activities Questionnaire (FAQ), Clinical Dementia Rating-Sum of Boxes (CDRSB) Alzheimer's Disease Assessment Scale-Cognitive Subscale 13 (ADAS13), Mini-Mental State Examination (MMSE), and Montreal Cognitive Assessment (MoCA).

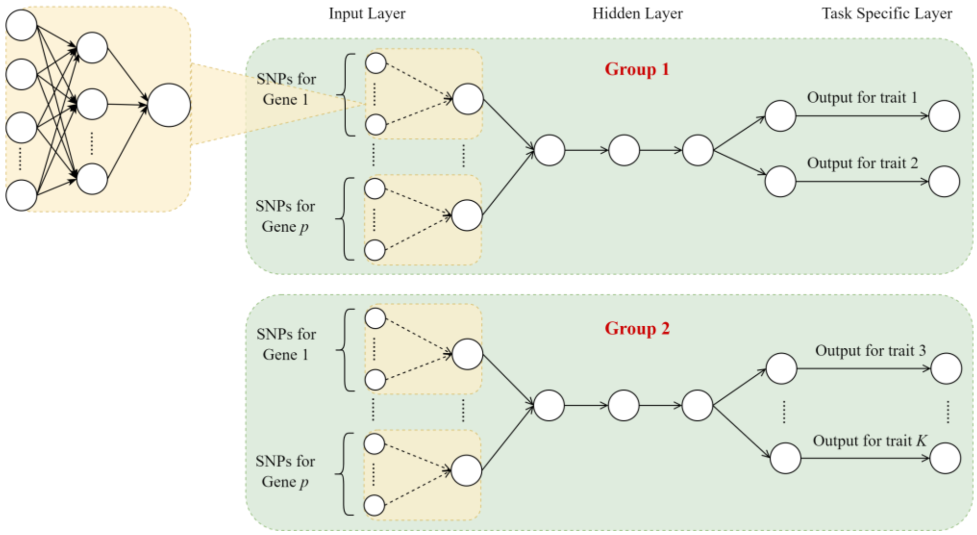

Supplementary Figure S3. Train separate hard parameter sharing models for each group of traits based on the affinity between traits. Suppose we have traits that are grouped into 2 groups.

1.2 Supplementary Tables

Supplementary Table S1. The details of simulation 1.

Sharing Situations	No. of underlying groups	Traits grouping[a]
Complete Sharing	1	(1, 2, 3, 4, 5, 6, 7)
Two-group Sharing	2	(1, 2, 3) and (4, 5, 6, 7)
Three-group Sharing	3	(1, 2, 3), (4, 5), and (6, 7)
Four-group Sharing	4	(1, 2), (3, 4), (5, 6) and (7)
No Sharing	7	(1), (2), (3), (4), (5), (6), and (7)

a. Traits in parathesis shared the same underlying causes.

Supplementary Table S2. Pearson correlations for seven traits under different numbers of underlying groups among traits. The effect size of causal variants is set such that the ratio $\sigma_\beta/\sigma_\epsilon$ = 1:3.

Sharing Situations	Different Strategies	Trait 1	Trait 2	Trait 3	Trait 4	Trait 5	Trait 6	Trait 7	Average Pearson of seven traits
Complete Sharing	Multi-Lasso	0.463	0.460	0.459	0.471	0.469	0.468	0.448	0.463
	HPS[a]	0.532	0.537	0.511	0.530	0.543	0.539	0.522	0.531
	2 Groups[b]	0.549	0.548	0.537	0.549	0.540	0.546	0.533	0.543
	3 Groups[c]	0.538	0.544	0.520	0.535	0.545	0.546	0.535	0.538
	4 Groups[d]	0.543	0.542	0.523	0.544	0.538	0.554	0.526	0.539
	STL[e]	0.440	0.448	0.425	0.449	0.425	0.421	0.424	0.433
Two-group Sharing	Multi-Lasso	0.427	0.436	0.438	0.420	0.427	0.408	0.422	0.425
	HPS	0.483	0.481	0.453	0.485	0.495	0.471	0.481	0.478
	2 Groups	0.485	0.496	0.477	0.505	0.507	0.495	0.501	0.495
	3 Groups	0.486	0.506	0.473	0.497	0.491	0.490	0.494	0.491
	4 Groups	0.488	0.500	0.472	0.501	0.501	0.492	0.507	0.494
	STL	0.430	0.414	0.413	0.339	0.323	0.352	0.371	0.377
Three-group Sharing	Multi-Lasso	0.428	0.428	0.435	0.402	0.396	0.358	0.379	0.404
	HPS	0.485	0.462	0.464	0.405	0.390	0.367	0.366	0.420
	2 Groups	0.482	0.482	0.488	0.442	0.423	0.402	0.416	0.448
	3 Groups	0.495	0.489	0.499	0.436	0.431	0.401	0.406	0.451
	4 Groups	0.489	0.479	0.494	0.454	0.416	0.401	0.399	0.447
	STL	0.444	0.455	0.443	0.386	0.404	0.391	0.390	0.416
Four-group Sharing	Multi-Lasso	0.436	0.440	0.386	0.386	0.394	0.377	0.394	0.402
	HPS	0.451	0.450	0.390	0.388	0.401	0.400	0.335	0.402
	2 Groups	0.476	0.455	0.426	0.421	0.420	0.408	0.310	0.417
	3 Groups	0.474	0.472	0.421	0.407	0.409	0.411	0.325	0.417
	4 Groups	0.469	0.473	0.405	0.422	0.420	0.418	0.345	0.422
	STL	0.407	0.445	0.436	0.395	0.400	0.356	0.346	0.398
No Sharing	Multi-Lasso	0.381	0.414	0.413	0.369	0.361	0.393	0.417	0.393
	HPS	0.320	0.332	0.375	0.286	0.300	0.296	0.345	0.322
	2 Groups	0.330	0.353	0.339	0.322	0.292	0.327	0.361	0.332
	3 Groups	0.316	0.328	0.358	0.312	0.299	0.320	0.381	0.331
	4 Groups	0.316	0.339	0.354	0.289	0.319	0.325	0.356	0.328
	STL	0.459	0.459	0.374	0.394	0.360	0.379	0.339	0.395

a. Hard parameter sharing across all layers except the last one.
b. The pre-specified number of branches for the auto-branch method is two.
c. The pre-specified number of branches for the auto-branch method is three.
d. The pre-specified number of branches for the auto-branch method is four.
e. Each trait is modeled independently without accounting for trait correlations.

Supplementary Table S3. Pearson correlations for seven traits under different numbers of underlying groups among traits. The effect size of causal variants is set such that the ratio $\sigma_\beta/\sigma_\epsilon$ = 1:4.

Sharing Situations	Different Strategies	Trait 1	Trait 2	Trait 3	Trait 4	Trait 5	Trait 6	Trait 7	Average Pearson of seven traits
Complete Sharing	Multi-Lasso	0.350	0.345	0.343	0.358	0.356	0.357	0.332	0.349
	HPS[a]	0.440	0.444	0.437	0.431	0.440	0.446	0.428	0.438
	2 Groups[b]	0.453	0.451	0.424	0.443	0.464	0.452	0.425	0.445
	3 Groups[c]	0.456	0.441	0.441	0.438	0.450	0.441	0.431	0.443
	4 Groups[d]	0.446	0.441	0.430	0.444	0.448	0.454	0.445	0.444
	STL[e]	0.340	0.341	0.348	0.330	0.328	0.366	0.346	0.343
Two-group Sharing	Multi-Lasso	0.311	0.321	0.323	0.311	0.318	0.297	0.313	0.313
	HPS	0.356	0.384	0.359	0.391	0.393	0.369	0.384	0.377
	2 Groups	0.388	0.399	0.375	0.407	0.401	0.388	0.397	0.394
	3 Groups	0.387	0.399	0.374	0.408	0.396	0.389	0.404	0.394
	4 Groups	0.375	0.389	0.376	0.409	0.405	0.387	0.400	0.392
	STL	0.358	0.348	0.333	0.295	0.285	0.289	0.325	0.319
Three-group Sharing	Multi-Lasso	0.307	0.307	0.316	0.293	0.286	0.252	0.276	0.291
	HPS	0.370	0.364	0.358	0.300	0.299	0.274	0.273	0.320
	2 Groups	0.392	0.383	0.381	0.304	0.327	0.297	0.282	0.338
	3 Groups	0.372	0.378	0.388	0.326	0.341	0.280	0.296	0.340
	4 Groups	0.385	0.377	0.392	0.317	0.301	0.292	0.292	0.337
	STL	0.316	0.326	0.329	0.286	0.289	0.230	0.257	0.290
Four-group Sharing	Multi-Lasso	0.321	0.323	0.278	0.277	0.292	0.273	0.287	0.293
	HPS	0.335	0.313	0.296	0.296	0.300	0.308	0.213	0.294
	2 Groups	0.356	0.349	0.301	0.309	0.311	0.318	0.226	0.310
	3 Groups	0.367	0.362	0.306	0.291	0.303	0.316	0.240	0.312
	4 Groups	0.368	0.364	0.311	0.304	0.320	0.313	0.212	0.313
	STL	0.337	0.358	0.276	0.282	0.260	0.314	0.276	0.300
No Sharing	Multi-Lasso	0.279	0.304	0.309	0.267	0.261	0.291	0.312	0.289
	HPS	0.208	0.220	0.268	0.189	0.199	0.206	0.251	0.220
	2 Groups	0.230	0.237	0.261	0.217	0.217	0.221	0.257	0.234
	3 Groups	0.229	0.214	0.251	0.244	0.203	0.248	0.259	0.235
	4 Groups	0.222	0.233	0.264	0.209	0.209	0.204	0.242	0.226
	STL	0.330	0.330	0.356	0.237	0.252	0.256	0.275	0.291

a. Hard parameter sharing across all layers except the last one.
b. The pre-specified number of branches for the auto-branch method is two.
c. The pre-specified number of branches for the auto-branch method is three.
d. The pre-specified number of branches for the auto-branch method is four.
e. Each trait is modeled independently without accounting for trait correlations.

Supplementary Table S4. Pearson correlations for seven traits under different numbers of underlying groups among traits. The effect size of causal variants is set such that the ratio $\sigma_\beta/\sigma_\epsilon$ = 1:5.

Sharing Situations	Different Strategies	Trait 1	Trait 2	Trait 3	Trait 4	Trait 5	Trait 6	Trait 7	Average Pearson of seven traits
Complete Sharing	Multi-Lasso	0.269	0.262	0.261	0.276	0.273	0.275	0.249	0.266
	HPS[a]	0.358	0.358	0.349	0.362	0.356	0.377	0.335	0.356
	2 Groups[b]	0.371	0.377	0.369	0.362	0.362	0.369	0.349	0.366
	3 Groups[c]	0.372	0.375	0.363	0.370	0.379	0.365	0.354	0.368
	4 Groups[d]	0.346	0.378	0.337	0.350	0.360	0.363	0.350	0.355
	STL[e]	0.262	0.261	0.268	0.240	0.266	0.269	0.246	0.259
Two-group Sharing	Multi-Lasso	0.233	0.249	0.246	0.252	0.239	0.220	0.235	0.239
	HPS	0.281	0.291	0.282	0.309	0.248	0.265	0.239	0.274
	2 Groups	0.314	0.316	0.309	0.346	0.292	0.284	0.274	0.305
	3 Groups	0.305	0.325	0.297	0.324	0.275	0.277	0.304	0.301
	4 Groups	0.316	0.337	0.309	0.332	0.296	0.271	0.264	0.304
	STL	0.265	0.276	0.256	0.275	0.222	0.243	0.244	0.254
Three-group Sharing	Multi-Lasso	0.224	0.223	0.235	0.219	0.212	0.182	0.209	0.215
	HPS	0.273	0.249	0.272	0.198	0.177	0.175	0.184	0.218
	2 Groups	0.298	0.304	0.308	0.239	0.220	0.206	0.185	0.251
	3 Groups	0.304	0.308	0.311	0.252	0.231	0.208	0.222	0.262
	4 Groups	0.304	0.286	0.293	0.234	0.246	0.211	0.195	0.253
	STL	0.256	0.267	0.283	0.217	0.233	0.151	0.193	0.229
Four-group Sharing	Multi-Lasso	0.243	0.243	0.206	0.204	0.225	0.204	0.215	0.220
	HPS	0.242	0.238	0.191	0.201	0.206	0.198	0.120	0.199
	2 Groups	0.287	0.270	0.235	0.204	0.233	0.224	0.142	0.228
	3 Groups	0.281	0.285	0.242	0.219	0.239	0.248	0.143	0.237
	4 Groups	0.284	0.279	0.221	0.219	0.225	0.240	0.174	0.235
	STL	0.273	0.293	0.226	0.251	0.192	0.196	0.179	0.230
No Sharing	Multi-Lasso	0.210	0.228	0.237	0.200	0.186	0.224	0.241	0.218
	HPS	0.124	0.141	0.146	0.137	0.132	0.131	0.170	0.140
	2 Groups	0.154	0.152	0.160	0.121	0.115	0.137	0.190	0.147
	3 Groups	0.146	0.116	0.169	0.126	0.132	0.119	0.159	0.138
	4 Groups	0.156	0.136	0.183	0.142	0.130	0.164	0.175	0.155
	STL	0.231	0.237	0.272	0.190	0.176	0.222	0.206	0.219

a. Hard parameter sharing across all layers except the last one.
b. The pre-specified number of branches for the auto-branch method is two.
c. The pre-specified number of branches for the auto-branch method is three.
d. The pre-specified number of branches for the auto-branch method is four.
e. Each trait is modeled independently without accounting for trait correlations.

Supplementary Table S5. Pearson correlations for seven traits under different numbers of underlying groups among traits. The effect size of causal variants is set such that the ratio $\sigma_\beta/\sigma_\epsilon$ = 1:6.

Sharing Situations	Different Strategies	Trait 1	Trait 2	Trait 3	Trait 4	Trait 5	Trait 6	Trait 7	Average Pearson of seven traits
Complete Sharing	Multi-Lasso	0.211	0.202	0.202	0.217	0.214	0.216	0.189	0.207
	HPS[a]	0.285	0.303	0.303	0.276	0.292	0.280	0.293	0.290
	2 Groups[b]	0.298	0.309	0.296	0.298	0.286	0.288	0.288	0.295
	3 Groups[c]	0.286	0.302	0.292	0.301	0.292	0.311	0.284	0.295
	4 Groups[d]	0.298	0.316	0.289	0.305	0.321	0.322	0.304	0.308
	STL[e]	0.190	0.200	0.209	0.218	0.225	0.226	0.201	0.210
Two-group Sharing	Multi-Lasso	0.176	0.188	0.188	0.185	0.191	0.170	0.187	0.183
	HPS	0.200	0.230	0.211	0.226	0.210	0.226	0.222	0.218
	2 Groups	0.192	0.228	0.235	0.262	0.262	0.243	0.253	0.239
	3 Groups	0.213	0.227	0.211	0.275	0.259	0.256	0.253	0.242
	4 Groups	0.204	0.233	0.204	0.277	0.249	0.221	0.254	0.235
	STL	0.200	0.227	0.190	0.161	0.181	0.167	0.176	0.186
Three-group Sharing	Multi-Lasso	0.166	0.166	0.179	0.167	0.161	0.135	0.163	0.163
	HPS	0.187	0.186	0.197	0.138	0.104	0.135	0.127	0.153
	2 Groups	0.213	0.230	0.230	0.182	0.175	0.156	0.160	0.192
	3 Groups	0.252	0.232	0.242	0.164	0.165	0.175	0.153	0.198
	4 Groups	0.225	0.207	0.224	0.186	0.179	0.143	0.166	0.190
	STL	0.211	0.203	0.191	0.169	0.171	0.126	0.145	0.174
Four-group Sharing	Multi-Lasso	0.188	0.187	0.156	0.155	0.177	0.157	0.166	0.169
	HPS	0.159	0.140	0.109	0.134	0.163	0.152	0.046	0.129
	2 Groups	0.203	0.202	0.145	0.142	0.151	0.168	0.101	0.159
	3 Groups	0.192	0.192	0.176	0.163	0.174	0.177	0.127	0.172
	4 Groups	0.210	0.210	0.168	0.163	0.183	0.173	0.114	0.174
	STL	0.210	0.258	0.151	0.141	0.158	0.171	0.120	0.173
No Sharing	Multi-Lasso	0.162	0.175	0.185	0.154	0.151	0.177	0.191	0.171
	HPS	0.077	0.077	0.099	0.046	0.054	0.071	0.120	0.078
	2 Groups	0.074	0.105	0.126	0.101	0.099	0.068	0.111	0.098
	3 Groups	0.057	0.082	0.110	0.064	0.101	0.111	0.094	0.088
	4 Groups	0.083	0.092	0.112	0.085	0.072	0.122	0.105	0.096
	STL	0.206	0.192	0.206	0.120	0.144	0.160	0.154	0.169

a. Hard parameter sharing across all layers except the last one.
b. The pre-specified number of branches for the auto-branch method is two.
c. The pre-specified number of branches for the auto-branch method is three.
d. The pre-specified number of branches for the auto-branch method is four.
e. Each trait is modeled independently without accounting for trait correlations.

Supplementary Table S6. RMSEs for seven traits under different numbers of underlying groups among traits. The effect size of causal variants is set such that the ratio $\sigma_\beta/\sigma_\epsilon$ = 1:3.

Sharing Situations	Different Strategies	Trait 1	Trait 2	Trait 3	Trait 4	Trait 5	Trait 6	Trait 7	Average Pearson of seven traits
Complete Sharing	Multi-Lasso	1.003	1.004	0.999	1.001	0.994	0.996	1.010	1.001
	HPS[a]	0.827	0.830	0.831	0.844	0.825	0.830	0.838	0.832
	2 Groups[b]	0.826	0.829	0.825	0.842	0.824	0.826	0.831	0.829
	3 Groups[c]	0.822	0.830	0.829	0.843	0.819	0.827	0.830	0.829
	4 Groups[d]	0.825	0.829	0.827	0.847	0.826	0.825	0.839	0.831
	STL[e]	0.904	0.899	0.902	0.898	0.890	0.897	0.889	0.897
Two-group Sharing	Multi-Lasso	1.030	1.026	1.031	1.041	1.030	1.046	1.048	1.036
	HPS	0.834	0.853	0.869	0.854	0.861	0.860	0.869	0.857
	2 Groups	0.826	0.847	0.858	0.850	0.857	0.857	0.862	0.851
	3 Groups	0.832	0.847	0.865	0.853	0.859	0.850	0.865	0.853
	4 Groups	0.827	0.842	0.857	0.847	0.855	0.856	0.864	0.850
	STL	0.883	0.880	0.886	0.921	0.902	0.906	0.916	0.899
Three-group Sharing	Multi-Lasso	1.041	1.030	1.033	1.049	1.050	1.095	1.081	1.054
	HPS	0.878	0.851	0.875	0.907	0.892	0.913	0.916	0.890
	2 Groups	0.863	0.836	0.860	0.890	0.888	0.906	0.902	0.878
	3 Groups	0.863	0.838	0.863	0.899	0.890	0.907	0.902	0.880
	4 Groups	0.861	0.846	0.856	0.896	0.886	0.906	0.904	0.879
	STL	0.900	0.881	0.880	0.926	0.893	0.933	0.930	0.906
Four-group Sharing	Multi-Lasso	1.024	1.019	1.071	1.071	1.064	1.078	1.061	1.056
	HPS	0.868	0.870	0.897	0.898	0.900	0.878	0.933	0.892
	2 Groups	0.853	0.866	0.902	0.897	0.886	0.873	0.922	0.886
	3 Groups	0.860	0.859	0.889	0.895	0.891	0.873	0.927	0.885
	4 Groups	0.857	0.860	0.895	0.897	0.893	0.876	0.928	0.887
	STL	0.878	0.857	0.928	0.914	0.914	0.905	0.935	0.904
No Sharing	Multi-Lasso	1.073	1.046	1.040	1.091	1.080	1.060	1.040	1.061
	HPS	0.935	0.947	0.938	0.950	0.948	0.925	0.945	0.941
	2 Groups	0.938	0.941	0.921	0.940	0.949	0.918	0.919	0.932
	3 Groups	0.938	0.949	0.933	0.946	0.943	0.917	0.928	0.936
	4 Groups	0.946	0.939	0.918	0.943	0.936	0.926	0.927	0.934
	STL	0.905	0.906	0.900	0.943	0.941	0.924	0.928	0.921

a. Hard parameter sharing across all layers except the last one.
b. The pre-specified number of branches for the auto-branch method is two.
c. The pre-specified number of branches for the auto-branch method is three.
d. The pre-specified number of branches for the auto-branch method is four.
e. Each trait is modeled independently without accounting for trait correlations.

Supplementary Table S7. RMSEs for seven traits under different numbers of underlying groups among traits. The effect size of causal variants is set such that the ratio $\sigma_\beta/\sigma_\epsilon$ = 1:4.

Sharing Situations	Different Strategies	Trait 1	Trait 2	Trait 3	Trait 4	Trait 5	Trait 6	Trait 7	Average Pearson of seven traits
Complete Sharing	Multi-Lasso	1.117	1.120	1.113	1.115	1.106	1.109	1.124	1.115
	HPS[a]	0.876	0.881	0.879	0.896	0.878	0.874	0.888	0.882
	2 Groups[b]	0.874	0.878	0.875	0.889	0.873	0.875	0.886	0.879
	3 Groups[c]	0.876	0.881	0.875	0.890	0.875	0.871	0.884	0.879
	4 Groups[d]	0.874	0.875	0.872	0.886	0.874	0.871	0.882	0.876
	STL[e]	0.950	0.949	0.943	0.943	0.926	0.933	0.917	0.937
Two-group Sharing	Multi-Lasso	1.143	1.140	1.143	1.149	1.136	1.153	1.156	1.145
	HPS	0.890	0.911	0.921	0.904	0.905	0.909	0.918	0.908
	2 Groups	0.877	0.898	0.905	0.900	0.903	0.906	0.911	0.900
	3 Groups	0.883	0.899	0.912	0.901	0.901	0.904	0.911	0.902
	4 Groups	0.879	0.897	0.908	0.897	0.903	0.903	0.910	0.900
	STL	0.936	0.927	0.938	0.965	0.940	0.941	0.959	0.944
Three-group Sharing	Multi-Lasso	1.157	1.145	1.148	1.154	1.156	1.194	1.179	1.162
	HPS	0.928	0.899	0.919	0.955	0.938	0.957	0.956	0.936
	2 Groups	0.911	0.892	0.914	0.950	0.933	0.957	0.955	0.930
	3 Groups	0.914	0.887	0.913	0.943	0.937	0.949	0.945	0.927
	4 Groups	0.915	0.889	0.909	0.947	0.934	0.953	0.943	0.927
	STL	0.955	0.944	0.932	0.967	0.927	0.970	0.964	0.951
Four-group Sharing	Multi-Lasso	1.138	1.132	1.175	1.178	1.162	1.178	1.164	1.161
	HPS	0.916	0.923	0.940	0.941	0.947	0.921	0.959	0.935
	2 Groups	0.908	0.916	0.939	0.936	0.933	0.918	0.963	0.930
	3 Groups	0.911	0.914	0.940	0.936	0.940	0.920	0.958	0.931
	4 Groups	0.901	0.913	0.937	0.939	0.933	0.919	0.964	0.929
	STL	0.933	0.909	0.961	0.947	0.949	0.950	0.967	0.945
No Sharing	Multi-Lasso	1.173	1.153	1.143	1.188	1.177	1.160	1.145	1.163
	HPS	0.983	0.994	0.975	0.977	0.986	0.964	0.975	0.979
	2 Groups	0.970	0.982	0.969	0.982	0.981	0.953	0.973	0.973
	3 Groups	0.976	0.986	0.971	0.973	0.975	0.957	0.973	0.973
	4 Groups	0.972	0.979	0.964	0.976	0.971	0.947	0.977	0.969
	STL	0.945	0.954	0.947	0.981	0.984	0.951	0.960	0.960

a. Hard parameter sharing across all layers except the last one.
b. The pre-specified number of branches for the auto-branch method is two.
c. The pre-specified number of branches for the auto-branch method is three.
d. The pre-specified number of branches for the auto-branch method is four.
e. Each trait is modeled independently without accounting for trait correlations.

Supplementary Table S8. RMSEs for seven traits under different numbers of underlying groups among traits. The effect size of causal variants is set such that the ratio $\sigma_\beta / \sigma_\epsilon$ = 1:5.

Sharing Situations	Different Strategies	Trait 1	Trait 2	Trait 3	Trait 4	Trait 5	Trait 6	Trait 7	Average Pearson of seven traits
Complete Sharing	Multi-Lasso	1.196	1.198	1.191	1.190	1.184	1.186	1.202	1.192
	HPS[a]	0.915	0.917	0.914	0.925	0.914	0.913	0.924	0.918
	2 Groups[b]	0.908	0.917	0.910	0.927	0.909	0.906	0.919	0.914
	3 Groups[c]	0.903	0.914	0.910	0.925	0.913	0.905	0.915	0.912
	4 Groups[d]	0.906	0.912	0.913	0.923	0.912	0.906	0.923	0.914
	STL[e]	0.986	0.979	0.973	0.966	0.958	0.960	0.947	0.967
Two-group Sharing	Multi-Lasso	1.215	1.211	1.215	1.205	1.208	1.223	1.228	1.215
	HPS	0.914	0.929	0.941	0.928	0.952	0.946	0.962	0.939
	2 Groups	0.907	0.926	0.932	0.921	0.943	0.945	0.955	0.933
	3 Groups	0.912	0.925	0.939	0.921	0.949	0.945	0.957	0.935
	4 Groups	0.910	0.921	0.934	0.922	0.945	0.947	0.955	0.933
	STL	0.972	0.960	0.967	0.971	0.979	0.972	0.977	0.971
Three-group Sharing	Multi-Lasso	1.233	1.220	1.223	1.223	1.225	1.256	1.241	1.232
	HPS	0.957	0.930	0.955	0.980	0.966	0.991	0.988	0.967
	2 Groups	0.947	0.921	0.948	0.979	0.956	0.977	0.975	0.958
	3 Groups	0.948	0.919	0.946	0.970	0.962	0.980	0.978	0.958
	4 Groups	0.953	0.923	0.950	0.974	0.965	0.982	0.977	0.961
	STL	0.988	0.976	0.960	0.996	0.952	0.982	0.980	0.976
Four-group Sharing	Multi-Lasso	1.212	1.207	1.242	1.246	1.225	1.242	1.231	1.230
	HPS	0.950	0.951	0.972	0.972	0.974	0.946	0.978	0.963
	2 Groups	0.941	0.951	0.965	0.958	0.964	0.942	0.978	0.957
	3 Groups	0.940	0.952	0.958	0.958	0.962	0.944	0.986	0.957
	4 Groups	0.939	0.954	0.968	0.968	0.960	0.944	0.972	0.958
	STL	0.971	0.947	0.995	0.987	0.978	0.970	0.997	0.978
No Sharing	Multi-Lasso	1.239	1.223	1.213	1.249	1.239	1.225	1.213	1.229
	HPS	0.998	1.004	0.988	0.999	0.996	0.984	1.001	0.995
	2 Groups	0.992	1.004	0.992	0.997	0.992	0.977	0.990	0.992
	3 Groups	0.995	1.011	0.998	1.002	0.997	0.970	0.992	0.995
	4 Groups	0.992	1.010	0.991	1.002	0.995	0.970	0.984	0.992
	STL	0.979	0.986	0.981	1.006	1.003	0.976	0.979	0.987

a. Hard parameter sharing across all layers except the last one.
b. The pre-specified number of branches for the auto-branch method is two.
c. The pre-specified number of branches for the auto-branch method is three.
d. The pre-specified number of branches for the auto-branch method is four.
e. Each trait is modeled independently without accounting for trait correlations.

Supplementary Table S9. RMSEs for seven traits under different numbers of underlying groups among traits. The effect size of causal variants is set such that the ratio $\sigma_{\beta}/\sigma_{\epsilon}$ = 1:6.

Sharing Situations	Different Strategies	Trait 1	Trait 2	Trait 3	Trait 4	Trait 5	Trait 6	Trait 7	Average Pearson of seven traits
Complete Sharing	Multi-Lasso	1.249	1.253	1.244	1.243	1.238	1.239	1.256	1.246
	HPS[a]	0.936	0.943	0.937	0.955	0.940	0.934	0.948	0.942
	2 Groups[b]	0.931	0.936	0.935	0.945	0.937	0.927	0.941	0.936
	3 Groups[c]	0.930	0.940	0.935	0.951	0.935	0.929	0.942	0.937
	4 Groups[d]	0.934	0.941	0.941	0.948	0.934	0.929	0.944	0.939
	STL[e]	1.013	1.005	1.005	0.982	0.979	0.987	0.966	0.991
Two-group Sharing	Multi-Lasso	1.267	1.266	1.266	1.267	1.252	1.269	1.273	1.266
	HPS	0.954	0.964	0.971	0.952	0.957	0.960	0.972	0.961
	2 Groups	0.944	0.963	0.973	0.947	0.955	0.958	0.969	0.958
	3 Groups	0.946	0.956	0.965	0.951	0.958	0.957	0.969	0.957
	4 Groups	0.949	0.961	0.970	0.944	0.953	0.954	0.967	0.957
	STL	1.002	0.988	1.002	1.007	0.991	0.982	0.997	0.995
Three-group Sharing	Multi-Lasso	1.283	1.271	1.273	1.270	1.272	1.298	1.282	1.278
	HPS	0.981	0.946	0.975	0.997	0.982	1.005	0.995	0.983
	2 Groups	0.974	0.948	0.972	0.995	0.980	0.994	0.987	0.979
	3 Groups	0.972	0.945	0.964	0.993	0.978	0.996	0.986	0.976
	4 Groups	0.973	0.945	0.965	0.993	0.979	0.997	0.985	0.977
	STL	1.016	0.996	0.990	1.013	0.977	1.004	0.994	0.999
Four-group Sharing	Multi-Lasso	1.264	1.258	1.287	1.292	1.268	1.286	1.276	1.276
	HPS	0.964	0.980	0.983	0.979	0.976	0.958	0.987	0.975
	2 Groups	0.965	0.973	0.976	0.978	0.975	0.956	0.988	0.973
	3 Groups	0.966	0.977	0.976	0.978	0.976	0.953	0.994	0.974
	4 Groups	0.965	0.972	0.980	0.975	0.978	0.959	0.986	0.974
	STL	0.996	0.973	1.011	1.006	0.986	0.981	1.008	0.994
No Sharing	Multi-Lasso	1.284	1.272	1.262	1.290	1.281	1.270	1.260	1.274
	HPS	0.998	1.017	1.004	1.006	0.997	0.987	1.001	1.001
	2 Groups	1.001	1.010	1.000	1.002	1.000	0.987	1.002	1.000
	3 Groups	0.993	1.011	1.010	1.007	1.001	0.986	1.000	1.001
	4 Groups	1.001	1.012	1.002	1.004	1.001	0.987	1.003	1.001
	STL	0.993	1.019	1.012	1.030	1.011	0.990	1.004	1.009

a. Hard parameter sharing across all layers except the last one.
b. The pre-specified number of branches for the auto-branch method is two.
c. The pre-specified number of branches for the auto-branch method is three.
d. The pre-specified number of branches for the auto-branch method is four.
e. Each trait is modeled independently without accounting for trait correlations.

Supplementary Table S10. Pearson correlations for seven traits as the relative contributions between unique causal factors and shared causal factors increases. The effect size of causal variants is set such that the ratio $(\sigma_\beta^2 + \sigma_{\beta_s}^2)/\sigma_\epsilon^2$ = 1:9, where β represents trait-specific effects and β_s represents shared effects among traits.

$\sigma_\beta^2/\sigma_{\beta_s}^2$	Different Strategies	Trait 1	Trait 2	Trait 3	Trait 4	Trait 5	Trait 6	Trait 7	Average Pearson of seven traits
1:9	Multi-Lasso	0.454	0.445	0.453	0.436	0.434	0.445	0.437	0.444
	HPS[a]	0.495	0.504	0.498	0.489	0.495	0.489	0.492	0.495
	2 Groups[b]	0.504	0.510	0.499	0.492	0.498	0.492	0.510	0.501
	3 Groups[c]	0.491	0.524	0.498	0.493	0.507	0.483	0.521	0.502
	4 Groups[d]	0.502	0.514	0.504	0.499	0.505	0.489	0.517	0.504
	STL[e]	0.414	0.422	0.409	0.413	0.417	0.430	0.410	0.416
3:7	Multi-Lasso	0.439	0.442	0.442	0.430	0.439	0.455	0.433	0.440
	HPS	0.434	0.438	0.423	0.436	0.416	0.418	0.432	0.428
	2 Groups	0.440	0.445	0.447	0.466	0.437	0.433	0.438	0.444
	3 Groups	0.441	0.457	0.435	0.473	0.441	0.455	0.450	0.450
	4 Groups	0.435	0.440	0.442	0.471	0.438	0.433	0.449	0.444
	STL	0.408	0.430	0.412	0.410	0.408	0.402	0.404	0.411
5:5	Multi-Lasso	0.439	0.430	0.439	0.434	0.407	0.429	0.423	0.429
	HPS	0.411	0.402	0.414	0.417	0.388	0.401	0.392	0.404
	2 Groups	0.416	0.411	0.415	0.447	0.419	0.420	0.401	0.418
	3 Groups	0.431	0.400	0.441	0.444	0.404	0.402	0.420	0.420
	4 Groups	0.418	0.395	0.418	0.428	0.411	0.422	0.407	0.414
	STL	0.435	0.412	0.417	0.393	0.392	0.366	0.387	0.400
7:3	Multi-Lasso	0.230	0.384	0.153	0.375	0.656	0.277	0.373	0.350
	HPS	0.380	0.392	0.389	0.381	0.392	0.389	0.405	0.390
	2 Groups	0.408	0.394	0.388	0.399	0.395	0.412	0.416	0.402
	3 Groups	0.390	0.395	0.407	0.400	0.407	0.413	0.412	0.403
	4 Groups	0.396	0.395	0.423	0.383	0.421	0.415	0.415	0.407
	STL	0.394	0.411	0.402	0.383	0.400	0.389	0.384	0.395
9:1	Multi-Lasso	0.193	0.560	0.498	0.246	0.358	0.409	0.233	0.357
	HPS	0.338	0.332	0.301	0.325	0.352	0.302	0.353	0.329
	2 Groups	0.336	0.355	0.326	0.352	0.379	0.319	0.379	0.349
	3 Groups	0.369	0.356	0.335	0.366	0.362	0.307	0.379	0.353
	4 Groups	0.347	0.346	0.329	0.345	0.368	0.303	0.398	0.348
	STL	0.421	0.396	0.357	0.366	0.364	0.337	0.341	0.369

a. Hard parameter sharing across all layers except the last one.
b. The pre-specified number of branches for the auto-branch method is two.
c. The pre-specified number of branches for the auto-branch method is three.
d. The pre-specified number of branches for the auto-branch method is four.
e. Each trait is modeled independently without accounting for trait correlations.

Supplementary Table S11. Pearson correlations for seven traits as the relative contributions between unique causal factors and shared causal factors increases. The effect size of causal variants is set such that the ratio $(\sigma_{\beta}^2 + \sigma_{\beta_s}^2)/\sigma_{\epsilon}^2$ = 1:16, where β represents trait-specific effects and β_s represents shared effects among traits.

$\sigma_{\beta}^2/\sigma_{\beta_s}^2$	Different Strategies	Trait 1	Trait 2	Trait 3	Trait 4	Trait 5	Trait 6	Trait 7	Average Pearson of seven traits
1:9	Multi-Lasso	0.323	0.324	0.325	0.310	0.328	0.334	0.323	0.324
	HPS[a]	0.405	0.390	0.384	0.390	0.418	0.395	0.396	0.397
	2 Groups[b]	0.407	0.406	0.392	0.407	0.405	0.414	0.402	0.405
	3 Groups[c]	0.421	0.419	0.400	0.424	0.426	0.425	0.410	0.418
	4 Groups[d]	0.419	0.429	0.407	0.421	0.420	0.415	0.403	0.416
	STL[e]	0.327	0.329	0.332	0.327	0.339	0.299	0.330	0.326
3:7	Multi-Lasso	0.298	0.296	0.292	0.323	0.300	0.316	0.316	0.306
	HPS	0.340	0.328	0.330	0.335	0.331	0.350	0.334	0.335
	2 Groups	0.348	0.345	0.348	0.324	0.343	0.355	0.350	0.345
	3 Groups	0.341	0.327	0.353	0.350	0.354	0.345	0.342	0.345
	4 Groups	0.346	0.337	0.348	0.331	0.336	0.350	0.354	0.343
	STL	0.291	0.334	0.303	0.307	0.256	0.286	0.301	0.297
5:5	Multi-Lasso	0.334	0.288	0.303	0.317	0.307	0.326	0.323	0.314
	HPS	0.288	0.268	0.288	0.312	0.290	0.291	0.320	0.294
	2 Groups	0.338	0.319	0.312	0.324	0.319	0.313	0.311	0.319
	3 Groups	0.318	0.300	0.298	0.334	0.296	0.332	0.317	0.314
	4 Groups	0.321	0.315	0.316	0.320	0.321	0.299	0.301	0.313
	STL	0.337	0.277	0.297	0.284	0.279	0.303	0.290	0.295
7:3	Multi-Lasso	0.311	0.274	0.293	0.296	0.327	0.278	0.312	0.299
	HPS	0.270	0.233	0.230	0.231	0.273	0.221	0.254	0.245
	2 Groups	0.273	0.248	0.246	0.280	0.301	0.246	0.269	0.266
	3 Groups	0.285	0.259	0.248	0.257	0.274	0.241	0.283	0.264
	4 Groups	0.296	0.258	0.253	0.272	0.295	0.244	0.277	0.271
	STL	0.335	0.277	0.259	0.300	0.269	0.247	0.273	0.280
9:1	Multi-Lasso	0.305	0.285	0.294	0.299	0.328	0.280	0.301	0.299
	HPS	0.240	0.227	0.245	0.251	0.243	0.220	0.215	0.234
	2 Groups	0.264	0.256	0.246	0.258	0.261	0.246	0.198	0.247
	3 Groups	0.256	0.254	0.242	0.256	0.259	0.253	0.228	0.250
	4 Groups	0.253	0.237	0.230	0.252	0.256	0.243	0.241	0.245
	STL	0.302	0.303	0.293	0.289	0.254	0.244	0.225	0.273

a. Hard parameter sharing across all layers except the last one.
b. The pre-specified number of branches for the auto-branch method is two.
c. The pre-specified number of branches for the auto-branch method is three.
d. The pre-specified number of branches for the auto-branch method is four.
e. Each trait is modeled independently without accounting for trait correlations.

Supplementary Table S12. Pearson correlations for seven traits as the relative contributions between unique causal factors and shared causal factors increases. The effect size of causal variants is set such that the ratio $(\sigma_\beta^2 + \sigma_{\beta_s}^2)/\sigma_\epsilon^2$ = 1:25, where β represents trait-specific effects and β_s represents shared effects among traits.

$\sigma_\beta^2/\sigma_{\beta_s}^2$	Different Strategies	Trait 1	Trait 2	Trait 3	Trait 4	Trait 5	Trait 6	Trait 7	Average Pearson of seven traits
1:9	Multi-Lasso	0.213	0.221	0.211	0.220	0.236	0.205	0.201	0.215
	HPS[a]	0.252	0.276	0.260	0.287	0.282	0.282	0.255	0.271
	2 Groups[b]	0.273	0.306	0.286	0.298	0.285	0.281	0.263	0.285
	3 Groups[c]	0.281	0.316	0.300	0.303	0.291	0.279	0.280	0.293
	4 Groups[d]	0.279	0.300	0.293	0.298	0.291	0.274	0.268	0.286
	STL[e]	0.234	0.248	0.245	0.237	0.235	0.211	0.214	0.232
3:7	Multi-Lasso	0.214	0.089	0.021	0.302	0.324	0.231	0.418	0.228
	HPS	0.263	0.268	0.253	0.272	0.281	0.262	0.276	0.268
	2 Groups	0.267	0.286	0.271	0.303	0.287	0.270	0.302	0.284
	3 Groups	0.273	0.297	0.273	0.291	0.292	0.265	0.275	0.281
	4 Groups	0.283	0.286	0.281	0.294	0.280	0.260	0.289	0.282
	STL	0.200	0.238	0.217	0.249	0.204	0.241	0.208	0.222
5:5	Multi-Lasso	0.287	0.376	-0.002	0.117	0.285	0.116	0.341	0.217
	HPS	0.211	0.225	0.219	0.214	0.224	0.231	0.237	0.223
	2 Groups	0.224	0.243	0.254	0.213	0.241	0.250	0.274	0.243
	3 Groups	0.235	0.237	0.258	0.214	0.232	0.233	0.256	0.238
	4 Groups	0.240	0.226	0.233	0.222	0.231	0.232	0.255	0.234
	STL	0.239	0.231	0.250	0.229	0.207	0.197	0.217	0.224
7:3	Multi-Lasso	0.242	0.235	0.225	0.183	0.205	0.221	0.186	0.214
	HPS	0.152	0.166	0.128	0.157	0.140	0.157	0.158	0.151
	2 Groups	0.182	0.188	0.147	0.173	0.172	0.183	0.193	0.177
	3 Groups	0.210	0.199	0.166	0.182	0.189	0.207	0.191	0.192
	4 Groups	0.199	0.189	0.172	0.183	0.195	0.181	0.197	0.188
	STL	0.243	0.248	0.227	0.173	0.178	0.172	0.178	0.203
9:1	Multi-Lasso	0.206	0.204	0.226	0.231	0.225	0.236	0.187	0.216
	HPS	0.121	0.135	0.136	0.148	0.129	0.148	0.122	0.134
	2 Groups	0.155	0.167	0.160	0.169	0.157	0.139	0.135	0.155
	3 Groups	0.167	0.186	0.156	0.178	0.158	0.167	0.113	0.161
	4 Groups	0.159	0.176	0.146	0.163	0.157	0.153	0.150	0.158
	STL	0.250	0.217	0.224	0.225	0.227	0.215	0.147	0.215

a. Hard parameter sharing across all layers except the last one.
b. The pre-specified number of branches for the auto-branch method is two.
c. The pre-specified number of branches for the auto-branch method is three.
d. The pre-specified number of branches for the auto-branch method is four.
e. Each trait is modeled independently without accounting for trait correlations.

Supplementary Table S13. Pearson correlations for seven traits as the relative contributions between unique causal factors and shared causal factors increases. The effect size of causal variants is set such that the ratio $(\sigma_{\beta}^2 + \sigma_{\beta_s}^2)/\sigma_{\epsilon}^2$ = 1:36, where β represents trait-specific effects and β_s represents shared effects among traits.

$\sigma_{\beta}^2/\sigma_{\beta_s}^2$	Different Strategies	Trait 1	Trait 2	Trait 3	Trait 4	Trait 5	Trait 6	Trait 7	Average Pearson of seven traits
1:9	Multi-Lasso	0.192	0.189	0.207	0.216	0.205	0.198	0.202	0.201
	HPS[a]	0.257	0.262	0.242	0.267	0.257	0.248	0.277	0.259
	2 Groups[b]	0.277	0.272	0.243	0.280	0.270	0.262	0.282	0.269
	3 Groups[c]	0.260	0.277	0.260	0.263	0.290	0.269	0.290	0.273
	4 Groups[d]	0.260	0.261	0.265	0.279	0.274	0.272	0.274	0.269
	STL[e]	0.195	0.200	0.194	0.229	0.186	0.214	0.182	0.200
3:7	Multi-Lasso	0.182	0.200	0.172	0.178	0.186	0.186	0.207	0.187
	HPS	0.206	0.197	0.212	0.210	0.212	0.208	0.241	0.212
	2 Groups	0.219	0.214	0.236	0.224	0.223	0.204	0.240	0.223
	3 Groups	0.206	0.208	0.246	0.214	0.237	0.225	0.252	0.227
	4 Groups	0.210	0.220	0.233	0.221	0.227	0.232	0.235	0.225
	STL	0.188	0.181	0.204	0.157	0.160	0.154	0.198	0.177
5:5	Multi-Lasso	0.175	0.177	0.189	0.181	0.172	0.178	0.162	0.176
	HPS	0.145	0.160	0.166	0.133	0.152	0.154	0.152	0.152
	2 Groups	0.183	0.174	0.178	0.179	0.180	0.178	0.168	0.177
	3 Groups	0.177	0.182	0.185	0.194	0.164	0.189	0.165	0.179
	4 Groups	0.175	0.189	0.189	0.213	0.168	0.169	0.150	0.179
	STL	0.195	0.170	0.202	0.151	0.156	0.156	0.163	0.170
7:3	Multi-Lasso	0.163	0.209	0.174	0.199	0.199	0.175	0.189	0.187
	HPS	0.121	0.132	0.124	0.132	0.161	0.119	0.130	0.131
	2 Groups	0.155	0.162	0.130	0.163	0.172	0.144	0.141	0.152
	3 Groups	0.136	0.162	0.149	0.161	0.160	0.139	0.159	0.152
	4 Groups	0.157	0.154	0.136	0.155	0.169	0.145	0.162	0.154
	STL	0.196	0.190	0.191	0.182	0.189	0.153	0.135	0.177
9:1	Multi-Lasso	0.178	0.154	0.169	0.192	0.170	0.159	0.158	0.168
	HPS	0.074	0.120	0.082	0.082	0.099	0.073	0.069	0.086
	2 Groups	0.097	0.097	0.087	0.115	0.128	0.107	0.118	0.107
	3 Groups	0.117	0.119	0.095	0.107	0.114	0.097	0.123	0.110
	4 Groups	0.094	0.106	0.090	0.134	0.117	0.133	0.103	0.111
	STL	0.177	0.179	0.160	0.170	0.148	0.132	0.119	0.155

a. Hard parameter sharing across all layers except the last one.
b. The pre-specified number of branches for the auto-branch method is two.
c. The pre-specified number of branches for the auto-branch method is three.
d. The pre-specified number of branches for the auto-branch method is four.
e. Each trait is modeled independently without accounting for trait correlations.

Supplementary Table S14. RMSEs for seven traits as the relative contributions between unique causal factors and shared causal factors increases. The effect size of causal variants is set such that the ratio $(\sigma_\beta^2 + \sigma_{\beta_s}^2)/\sigma_\epsilon^2$ = 1:9, where β represents trait-specific effects and β_s represents shared effects among traits.

$\sigma_\beta^2/\sigma_{\beta_s}^2$	Different Strategies	Trait 1	Trait 2	Trait 3	Trait 4	Trait 5	Trait 6	Trait 7	Average Pearson of seven traits
1:9	Multi-Lasso	1.012	1.031	1.009	1.024	1.024	1.016	1.020	1.019
	HPS[a]	0.850	0.853	0.868	0.850	0.859	0.849	0.838	0.852
	2 Groups[b]	0.850	0.851	0.865	0.847	0.854	0.853	0.837	0.851
	3 Groups[c]	0.847	0.847	0.868	0.848	0.860	0.853	0.831	0.851
	4 Groups[d]	0.846	0.848	0.861	0.849	0.858	0.850	0.833	0.849
	STL[e]	0.899	0.902	0.884	0.897	0.899	0.883	0.889	0.893
3:7	Multi-Lasso	1.023	1.031	1.025	1.042	1.031	1.013	1.014	1.028
	HPS	0.887	0.899	0.889	0.883	0.880	0.877	0.884	0.886
	2 Groups	0.883	0.897	0.883	0.875	0.881	0.873	0.880	0.882
	3 Groups	0.876	0.900	0.888	0.877	0.873	0.877	0.884	0.882
	4 Groups	0.882	0.899	0.887	0.876	0.874	0.873	0.884	0.882
	STL	0.910	0.915	0.913	0.908	0.906	0.907	0.920	0.910
5:5	Multi-Lasso	1.028	1.038	1.038	1.037	1.060	1.040	1.040	1.040
	HPS	0.897	0.914	0.901	0.906	0.900	0.912	0.920	0.905
	2 Groups	0.888	0.907	0.892	0.904	0.896	0.912	0.921	0.900
	3 Groups	0.891	0.916	0.899	0.909	0.900	0.902	0.913	0.903
	4 Groups	0.890	0.905	0.896	0.900	0.893	0.911	0.917	0.899
	STL	0.901	0.908	0.905	0.909	0.909	0.917	0.911	0.908
7:3	Multi-Lasso	1.057	1.044	1.043	1.022	1.035	1.026	1.018	1.038
	HPS	0.920	0.921	0.917	0.924	0.923	0.900	0.915	0.918
	2 Groups	0.909	0.918	0.913	0.920	0.918	0.892	0.903	0.912
	3 Groups	0.914	0.926	0.909	0.918	0.918	0.887	0.906	0.912
	4 Groups	0.910	0.917	0.913	0.928	0.916	0.891	0.904	0.913
	STL	0.912	0.912	0.904	0.936	0.903	0.908	0.892	0.913
9:1	Multi-Lasso	1.054	1.037	1.074	1.041	1.047	1.093	1.032	1.054
	HPS	0.927	0.932	0.937	0.933	0.924	0.946	0.942	0.933
	2 Groups	0.925	0.920	0.931	0.928	0.909	0.940	0.933	0.926
	3 Groups	0.929	0.915	0.929	0.923	0.919	0.950	0.934	0.928
	4 Groups	0.933	0.914	0.931	0.920	0.914	0.946	0.925	0.926
	STL	0.895	0.901	0.922	0.929	0.907	0.945	0.940	0.917

a. Hard parameter sharing across all layers except the last one.
b. The pre-specified number of branches for the auto-branch method is two.
c. The pre-specified number of branches for the auto-branch method is three.
d. The pre-specified number of branches for the auto-branch method is four.
e. Each trait is modeled independently without accounting for trait correlations.

Supplementary Table S15. RMSEs for seven traits as the relative contributions between unique causal factors and shared causal factors increases. The effect size of causal variants is set such that the ratio $(\sigma_\beta^2 + \sigma_{\beta_s}^2)/\sigma_\epsilon^2$ = 1:16, where β represents trait-specific effects and β_s represents shared effects among traits.

$\sigma_\beta^2/\sigma_{\beta_s}^2$	Different Strategies	Trait 1	Trait 2	Trait 3	Trait 4	Trait 5	Trait 6	Trait 7	Average Pearson of seven traits
1:9	Multi-Lasso	1.137	1.142	1.149	1.155	1.138	1.125	1.143	1.141
	HPS[a]	0.912	0.907	0.894	0.903	0.890	0.886	0.903	0.899
	2 Groups[b]	0.906	0.907	0.894	0.899	0.895	0.887	0.903	0.899
	3 Groups[c]	0.903	0.902	0.891	0.893	0.892	0.881	0.896	0.894
	4 Groups[d]	0.909	0.908	0.896	0.899	0.889	0.883	0.902	0.898
	STL[e]	0.937	0.926	0.937	0.954	0.938	0.928	0.939	0.937
3:7	Multi-Lasso	1.158	1.156	1.154	1.136	1.156	1.144	1.143	1.149
	HPS	0.936	0.932	0.921	0.935	0.940	0.931	0.917	0.930
	2 Groups	0.929	0.930	0.917	0.924	0.934	0.922	0.919	0.925
	3 Groups	0.931	0.925	0.913	0.923	0.929	0.922	0.918	0.923
	4 Groups	0.937	0.928	0.913	0.924	0.926	0.924	0.916	0.924
	STL	0.966	0.956	0.943	0.941	0.941	0.943	0.940	0.947
5:5	Multi-Lasso	1.129	1.161	1.148	1.146	1.154	1.149	1.137	1.146
	HPS	0.939	0.969	0.946	0.958	0.940	0.955	0.944	0.950
	2 Groups	0.937	0.959	0.943	0.945	0.939	0.947	0.938	0.944
	3 Groups	0.932	0.957	0.942	0.948	0.939	0.947	0.946	0.944
	4 Groups	0.940	0.968	0.946	0.952	0.939	0.944	0.945	0.948
	STL	0.950	0.961	0.968	0.959	0.949	0.971	0.961	0.960
7:3	Multi-Lasso	1.143	1.173	1.160	1.154	1.138	1.168	1.148	1.155
	HPS	0.971	0.970	0.967	0.954	0.957	0.970	0.965	0.965
	2 Groups	0.964	0.972	0.959	0.945	0.955	0.970	0.960	0.961
	3 Groups	0.967	0.970	0.964	0.947	0.956	0.966	0.958	0.961
	4 Groups	0.970	0.971	0.956	0.946	0.956	0.962	0.961	0.960
	STL	0.968	0.953	0.955	0.955	0.958	0.974	0.980	0.963
9:1	Multi-Lasso	1.163	1.157	1.162	1.162	1.133	1.170	1.145	1.156
	HPS	0.970	0.962	0.972	0.977	0.979	0.989	0.949	0.971
	2 Groups	0.972	0.960	0.968	0.968	0.972	0.973	0.946	0.966
	3 Groups	0.963	0.964	0.959	0.969	0.970	0.964	0.941	0.961
	4 Groups	0.968	0.960	0.976	0.974	0.979	0.971	0.944	0.967
	STL	0.945	0.952	0.941	0.952	0.968	0.974	0.973	0.958

a. Hard parameter sharing across all layers except the last one.
b. The pre-specified number of branches for the auto-branch method is two.
c. The pre-specified number of branches for the auto-branch method is three.
d. The pre-specified number of branches for the auto-branch method is four.
e. Each trait is modeled independently without accounting for trait correlations.

Supplementary Table S16. RMSEs for seven traits as the relative contributions between unique causal factors and shared causal factors increases. The effect size of causal variants is set such that the ratio $(\sigma_\beta^2 + \sigma_{\beta_s}^2)/\sigma_\epsilon^2$ = 1:25, where β represents trait-specific effects and β_s represents shared effects among traits.

$\sigma_\beta^2/\sigma_{\beta_s}^2$	Different Strategies	Trait 1	Trait 2	Trait 3	Trait 4	Trait 5	Trait 6	Trait 7	Average Pearson of seven traits
1:9	Multi-Lasso	1.245	1.238	1.234	1.244	1.220	1.240	1.250	1.239
	HPS[a]	0.939	0.940	0.930	0.930	0.931	0.925	0.948	0.935
	2 Groups[b]	0.937	0.939	0.930	0.932	0.929	0.923	0.942	0.933
	3 Groups[c]	0.934	0.936	0.933	0.933	0.928	0.917	0.947	0.933
	4 Groups[d]	0.939	0.942	0.927	0.931	0.928	0.919	0.943	0.933
	STL[e]	0.999	0.987	0.954	0.989	0.971	0.956	0.976	0.976
3:7	Multi-Lasso	1.225	1.222	1.215	1.236	1.201	1.210	1.241	1.222
	HPS	0.975	0.949	0.940	0.953	0.941	0.960	0.952	0.953
	2 Groups	0.965	0.943	0.936	0.952	0.934	0.955	0.948	0.948
	3 Groups	0.967	0.942	0.936	0.948	0.936	0.961	0.943	0.948
	4 Groups	0.965	0.944	0.941	0.955	0.943	0.960	0.948	0.951
	STL	0.986	0.994	0.975	0.991	0.977	0.984	0.995	0.986
5:5	Multi-Lasso	1.225	1.230	1.223	1.222	1.223	1.202	1.190	1.216
	HPS	0.960	0.960	0.950	0.966	0.985	0.953	0.956	0.961
	2 Groups	0.954	0.955	0.947	0.967	0.981	0.953	0.953	0.959
	3 Groups	0.957	0.958	0.949	0.975	0.977	0.956	0.944	0.959
	4 Groups	0.962	0.961	0.950	0.961	0.978	0.953	0.946	0.959
	STL	0.979	0.973	0.973	0.961	0.979	0.968	0.986	0.974
7:3	Multi-Lasso	1.214	1.205	1.232	1.265	1.244	1.224	1.227	1.230
	HPS	0.972	0.978	0.975	0.980	0.981	0.974	0.989	0.978
	2 Groups	0.972	0.973	0.974	0.973	0.983	0.970	0.979	0.975
	3 Groups	0.964	0.971	0.978	0.976	0.982	0.969	0.986	0.975
	4 Groups	0.971	0.972	0.979	0.980	0.987	0.972	0.977	0.977
	STL	0.973	0.971	0.995	1.003	1.005	0.992	0.991	0.990
9:1	Multi-Lasso	1.239	1.246	1.220	1.218	1.225	1.216	1.250	1.231
	HPS	0.997	0.971	0.995	0.986	0.993	0.997	0.995	0.990
	2 Groups	1.002	0.964	0.992	0.983	0.986	0.997	0.996	0.989
	3 Groups	0.997	0.964	0.991	0.984	0.984	0.998	0.995	0.988
	4 Groups	0.999	0.970	0.999	0.993	0.984	0.998	0.999	0.992
	STL	0.983	0.977	0.991	0.989	0.982	0.980	0.994	0.985

a. Hard parameter sharing across all layers except the last one.
b. The pre-specified number of branches for the auto-branch method is two.
c. The pre-specified number of branches for the auto-branch method is three.
d. The pre-specified number of branches for the auto-branch method is four.
e. Each trait is modeled independently without accounting for trait correlations.

Supplementary Table S17. RMSEs for seven traits as the relative contributions between unique causal factors and shared causal factors increases. The effect size of causal variants is set such that the ratio $(\sigma_{\beta}^2 + \sigma_{\beta_s}^2)/\sigma_{\epsilon}^2$ = 1:36, where β represents trait-specific effects and β_s represents shared effects among traits.

$\sigma_{\beta}^2/\sigma_{\beta_s}^2$	Different Strategies	Trait 1	Trait 2	Trait 3	Trait 4	Trait 5	Trait 6	Trait 7	Average Pearson of seven traits
1:9	Multi-Lasso	1.263	1.259	1.252	1.239	1.256	1.262	1.249	1.254
	HPS[a]	0.950	0.949	0.962	0.960	0.956	0.946	0.944	0.952
	2 Groups[b]	0.946	0.949	0.964	0.958	0.954	0.947	0.937	0.951
	3 Groups[c]	0.943	0.944	0.959	0.959	0.953	0.942	0.937	0.948
	4 Groups[d]	0.944	0.940	0.959	0.957	0.954	0.942	0.930	0.947
	STL[e]	1.002	0.987	1.005	0.996	0.982	0.990	0.991	0.993
3:7	Multi-Lasso	1.271	1.260	1.274	1.261	1.272	1.254	1.248	1.263
	HPS	0.949	0.962	0.961	0.958	0.966	0.951	0.972	0.960
	2 Groups	0.945	0.959	0.960	0.955	0.964	0.946	0.968	0.957
	3 Groups	0.949	0.958	0.958	0.956	0.965	0.945	0.968	0.957
	4 Groups	0.947	0.958	0.957	0.962	0.962	0.945	0.969	0.957
	STL	0.984	0.997	1.012	1.010	1.006	0.979	1.001	0.998
5:5	Multi-Lasso	1.276	1.269	1.269	1.273	1.268	1.266	1.277	1.271
	HPS	0.985	0.986	0.963	0.970	0.972	0.977	0.975	0.975
	2 Groups	0.984	0.980	0.966	0.970	0.967	0.981	0.975	0.975
	3 Groups	0.979	0.982	0.962	0.971	0.971	0.978	0.971	0.973
	4 Groups	0.979	0.983	0.962	0.965	0.966	0.982	0.972	0.973
	STL	0.988	1.008	0.986	0.995	1.020	1.006	0.995	1.000
7:3	Multi-Lasso	1.278	1.239	1.264	1.254	1.242	1.266	1.253	1.257
	HPS	0.989	0.978	0.978	0.997	0.982	0.979	0.973	0.982
	2 Groups	0.985	0.973	0.974	0.994	0.976	0.980	0.973	0.979
	3 Groups	0.987	0.977	0.977	0.991	0.979	0.982	0.975	0.981
	4 Groups	0.981	0.966	0.973	0.993	0.979	0.982	0.972	0.978
	STL	1.007	0.997	0.998	1.003	0.991	0.995	0.985	0.997
9:1	Multi-Lasso	1.274	1.276	1.265	1.250	1.277	1.282	1.281	1.272
	HPS	1.001	0.986	0.996	0.994	1.003	0.981	1.004	0.995
	2 Groups	1.001	0.987	1.000	0.987	1.000	0.981	1.002	0.994
	3 Groups	1.000	0.985	0.994	0.988	1.000	0.980	1.002	0.993
	4 Groups	1.001	0.985	0.993	0.986	1.005	0.981	1.007	0.994
	STL	1.001	0.994	0.991	1.017	0.996	1.007	1.021	1.004

a. Hard parameter sharing across all layers except the last one.
b. The pre-specified number of branches for the auto-branch method is two.
c. The pre-specified number of branches for the auto-branch method is three.
d. The pre-specified number of branches for the auto-branch method is four.
e. Each trait is modeled independently without accounting for trait correlations.

Supplementary Table S18. The summary of selection for genes used in real data analysis.

Gene	Chromosome	Start	End	No. SNP
COL11A1	1	103342022	103574052	156
FCER1G	1	161185086	161189038	7
GBP2	1	89571815	89591842	5
HSD11B1	1	209859524	209908295	17
PARP1	1	226548391	226595801	22
POU2F1	1	167190065	167396582	34
NGF	1	115828536	115880857	49
LHCGR	2	48913912	48982880	103
LRP2	2	169983618	170219122	180
APOD	3	195295572	195311076	20
SST	3	187386693	187388201	3
ALB	4	74269971	74287129	6
COL25A1	4	109731876	110223799	288
ADRB2	5	148206155	148208197	6
ARSB	5	78073036	78282357	179
FGF1	5	141971742	142077635	112
FGF10	5	44305096	44388784	12
FGF10-AS1	5	44388833	44414091	16
FGF18	5	170846666	170884630	31
NDUFS4	5	52856464	52979171	89
PPP2R2B-IT1	5	146293769	146299069	4
HSPA1A	6	31783290	31785719	2
MICA	6	31367560	31383092	71
MICAL1	6	109765265	109787171	8
CASC14	6	22134830	22147422	12
TBP	6	170863420	170881958	10
TBPL1	6	134273307	134308638	7
TREM2	6	41126243	41130924	0
CAV1	7	116164838	116201239	21
PON3	7	94989183	95025687	10
RELN	7	103112230	103629963	438
ADAM9	8	38854504	38962779	43
NAT1	8	18027970	18081198	89
NRG1	8	31497267	32622558	723
DFNB31	9	117164359	117267736	70
HSPA5	9	127997126	128003666	2
POMT1	9	134378288	134399193	9
RXRA	9	137218308	137332432	82
TLR4	9	120466452	120479769	12

CACNB2	10	18429605	18830688	522
MINPP1	10	89264222	89313218	15
TET1	10	70320116	70454239	70
APOC3	11	116700623	116703787	10
HBG2	11	5274420	5276011	2
ATF7	12	53901639	54020199	31
ATF7IP	12	14518565	14655869	50
SLC11A2	12	51373565	51422058	18
KLF5	13	73629113	73651680	18
HNRNPC	14	21677295	21737638	18
MTHFD1	14	64854758	64926725	46
PNP	14	20937537	20946165	10
APOC1	19	45417920	45422606	8
APOE	19	45409038	45412650	5
APOC1P1	19	45430059	45434643	4
APOC2	19	45449238	45452822	4
APOC4	19	45445494	45448753	4
TOMM40	19	45394476	45406946	14

Supplementary Table S19. P-value from Wilcoxon signed-rank test results for differences in Pearson correlations between methods across phenotypes.

	FDG	AV45	FAQ	CDRSB	ADAS13	MMSE	MoCA
	Compared to Multi-branch method with 2 group						
Multi_Lasso[a]	0.046*	0.001**	0.000**	0.139	0.959	0.327	0.901
HPS[b]	0.610	0.011*	0.053	0.021*	0.090	0.075	0.671
STL[c]	0.000**	0.014*	0.023*	0.104	0.277	0.025*	0.596
	Compared to Multi-branch method with 3 group						
Multi_Lasso	0.005**	0.000**	0.001**	0.097	0.721	0.695	0.773
HPS	0.084	0.010*	0.126	0.028*	0.039*	0.254	0.799
STL	0.000**	0.009**	0.069	0.132	0.172	0.142	0.897
	Compared to Multi-branch method with 4 group						
Multi_Lasso	0.003**	0.002**	0.000**	0.261	0.775	0.650	0.628
HPS	0.076	0.004**	0.051	0.090	0.040*	0.222	0.681
STL	0.000**	0.012*	0.008**	0.139	0.180	0.120	0.904

a. Multi_Lasso represent comparisons between Multi-Lasso models and auto-branch models.

b. HPS represent comparisons between hard-sharing models (shared across all layers except the last) and auto-branch models.

c. STL represent comparisons between single-task learning models (where each trait is modeled independently without considering inter-trait correlations) and auto-branch models.

$p < 0.05$ is considered statistically significant (*), and $p < 0.01$ is considered highly significant (**).

Supplementary Table S20. Probability of the gene being significantly predictive at 5% level. The pre-specified number of branches for the proposed auto-branch method is two.

Gene	Seven Alzheimer's related phenotypes[a]						
	FDG	AV45	FAQ	CDRSB	ADAS13	MMSE	MoCA
COL11A1	0.49	0.34	0.47	0.42	0.54	0.50	0.58
FCER1G	0.31	0.35	0.41	0.34	0.33	0.36	0.42
GBP2	0.47	0.44	0.54	0.64	0.52	0.51	0.46
HSD11B1	0.28	0.41	0.40	0.43	0.39	0.47	0.47
PARP1	0.44	0.39	0.51	0.52	0.63	0.57	0.54
POU2F1	0.57	0.43	0.39	0.36	0.36	0.52	0.40
NGF	0.39	0.52	0.40	0.54	0.43	0.52	0.50
LHCGR	0.37	0.34	0.37	0.44	0.39	0.44	0.45
LRP2	0.47	0.47	0.46	0.50	0.43	0.48	0.48
APOD	0.34	0.47	0.38	0.47	0.44	0.42	0.43
SST	0.36	0.36	0.45	0.45	0.33	0.39	0.34
ALB	0.47	0.45	0.40	0.33	0.41	0.49	0.37
COL25A1	0.33	0.52	0.53	0.46	0.44	0.47	0.48
ADRB2	0.53	0.35	0.48	0.32	0.48	0.41	0.40
ARSB	0.30	0.45	0.40	0.32	0.37	0.44	0.51
FGF1	0.55	0.56	0.71	0.68	0.56	0.60	0.56
FGF10	0.54	0.42	0.51	0.38	0.36	0.45	0.50
FGF10-AS1	0.38	0.46	0.44	0.33	0.39	0.52	0.51
FGF18	0.45	0.38	0.39	0.49	0.45	0.47	0.45
NDUFS4	0.54	0.63	0.47	0.42	0.33	0.46	0.55
PPP2R2B-IT1	0.47	0.54	0.47	0.45	0.51	0.48	0.53
HSPA1A	0.48	0.47	0.48	0.44	0.48	0.58	0.52
MICA	0.51	0.46	0.40	0.36	0.44	0.52	0.56
MICAL1	0.41	0.50	0.35	0.37	0.48	0.47	0.46
CASC14	0.26	0.33	0.27	0.36	0.36	0.38	0.44
TBP	0.52	0.38	0.46	0.32	0.50	0.48	0.43
TBPL1	0.51	0.53	0.53	0.45	0.48	0.49	0.45
CAV1	0.44	0.36	0.47	0.45	0.44	0.42	0.38
PON3	0.62	0.55	0.49	0.41	0.55	0.49	0.57
RELN	0.47	0.46	0.47	0.53	0.55	0.66	0.64
ADAM9	0.37	0.46	0.48	0.39	0.39	0.40	0.50
NAT1	0.42	0.46	0.43	0.45	0.60	0.48	0.49
NRG1	0.33	0.40	0.50	0.45	0.47	0.37	0.40
DFNB31	0.40	0.37	0.44	0.36	0.39	0.39	0.37
HSPA5	0.51	0.50	0.53	0.39	0.55	0.54	0.52
POMT1	0.45	0.39	0.38	0.39	0.48	0.38	0.45
RXRA	0.40	0.48	0.51	0.40	0.26	0.40	0.36
TLR4	0.35	0.36	0.39	0.37	0.41	0.44	0.34
CACNB2	0.42	0.37	0.43	0.35	0.41	0.47	0.41

MINPP1	0.39	0.47	0.43	0.40	0.43	0.58	0.56
TET1	0.38	0.37	0.58	0.49	0.49	0.50	0.40
APOC3	0.44	0.57	0.50	0.53	0.42	0.59	0.36
HBG2	0.48	0.41	0.42	0.42	0.47	0.45	0.47
ATF7	0.48	0.45	0.51	0.30	0.44	0.40	0.45
ATF7IP	0.51	0.53	0.47	0.46	0.41	0.41	0.44
SLC11A2	0.38	0.33	0.50	0.45	0.33	0.41	0.54
KLF5	0.33	0.37	0.51	0.44	0.38	0.44	0.33
HNRNPC	0.33	0.40	0.33	0.40	0.36	0.35	0.30
MTHFD1	0.60	0.42	0.50	0.45	0.44	0.45	0.47
PNP	0.62	0.46	0.46	0.49	0.58	0.41	0.50
APOC1	0.92	0.98	0.87	0.87	0.85	0.85	0.74
APOE	0.94	0.97	0.86	0.87	0.90	0.85	0.77
APOC1P1	0.70	0.62	0.71	0.66	0.62	0.62	0.64
APOC2	0.32	0.30	0.57	0.51	0.54	0.61	0.42
APOC4	0.31	0.32	0.52	0.51	0.47	0.51	0.39
TOMM40	0.85	0.93	0.79	0.82	0.77	0.83	0.73

a. Phenotypes include fluorodeoxyglucose (FDG) and florbetapir (AV45) PET imaging, Functional Activities Questionnaire (FAQ), Clinical Dementia Rating-Sum of Boxes (CDRSB) Alzheimer's Disease Assessment Scale-Cognitive Subscale 13 (ADAS13), Mini-Mental State Examination (MMSE), and Montreal Cognitive Assessment (MoCA).

Supplementary Table S21. Probability of the gene being significantly predictive at 5% level. The pre-specified number of branches for the proposed auto-branch method is three.

**Gene**	**Seven Alzheimer's related phenotypes**[a]						
	FDG	AV45	FAQ	CDRSB	ADAS13	MMSE	MoCA
COL11A1	0.53	0.36	0.49	0.43	0.47	0.54	0.41
FCER1G	0.41	0.31	0.34	0.33	0.42	0.34	0.38
GBP2	0.44	0.47	0.55	0.46	0.49	0.44	0.47
HSD11B1	0.41	0.49	0.41	0.43	0.43	0.42	0.42
PARP1	0.48	0.43	0.59	0.50	0.57	0.54	0.51
POU2F1	0.62	0.47	0.36	0.41	0.47	0.53	0.47
NGF	0.47	0.51	0.42	0.55	0.51	0.40	0.54
LHCGR	0.43	0.37	0.47	0.38	0.36	0.40	0.43
LRP2	0.43	0.49	0.39	0.47	0.43	0.53	0.41
APOD	0.33	0.38	0.44	0.46	0.49	0.46	0.37
SST	0.45	0.40	0.49	0.42	0.35	0.32	0.37
ALB	0.45	0.50	0.43	0.47	0.46	0.40	0.44
COL25A1	0.39	0.46	0.46	0.43	0.43	0.42	0.53
ADRB2	0.58	0.37	0.51	0.48	0.46	0.35	0.39
ARSB	0.46	0.50	0.35	0.38	0.43	0.41	0.43
FGF1	0.56	0.57	0.61	0.63	0.65	0.58	0.53
FGF10	0.48	0.41	0.39	0.47	0.42	0.45	0.55
FGF10-AS1	0.45	0.38	0.45	0.32	0.48	0.53	0.45
FGF18	0.44	0.30	0.48	0.55	0.50	0.51	0.48
NDUFS4	0.55	0.62	0.52	0.41	0.40	0.46	0.50
PPP2R2B-IT1	0.43	0.53	0.42	0.46	0.48	0.48	0.55
HSPA1A	0.46	0.51	0.41	0.40	0.53	0.48	0.48
MICA	0.45	0.45	0.35	0.33	0.36	0.45	0.47
MICAL1	0.41	0.49	0.44	0.45	0.57	0.45	0.62
CASC14	0.38	0.40	0.38	0.35	0.36	0.39	0.46
TBP	0.57	0.49	0.53	0.46	0.50	0.51	0.39
TBPL1	0.54	0.53	0.59	0.47	0.58	0.46	0.52
CAV1	0.50	0.36	0.44	0.42	0.43	0.40	0.35
PON3	0.64	0.58	0.57	0.52	0.58	0.48	0.48
RELN	0.53	0.44	0.49	0.57	0.61	0.63	0.57
ADAM9	0.38	0.51	0.45	0.41	0.43	0.56	0.39
NAT1	0.39	0.47	0.45	0.49	0.51	0.48	0.55
NRG1	0.43	0.41	0.47	0.44	0.47	0.43	0.45
DFNB31	0.35	0.36	0.41	0.34	0.33	0.42	0.37
HSPA5	0.54	0.49	0.52	0.47	0.68	0.53	0.50
POMT1	0.43	0.36	0.43	0.38	0.42	0.38	0.37
RXRA	0.41	0.49	0.49	0.47	0.38	0.50	0.36
TLR4	0.37	0.45	0.46	0.38	0.36	0.41	0.50
CACNB2	0.51	0.34	0.39	0.39	0.46	0.52	0.45

MINPP1	0.38	0.47	0.46	0.36	0.45	0.53	0.53
TET1	0.42	0.41	0.45	0.47	0.44	0.49	0.34
APOC3	0.50	0.46	0.37	0.51	0.37	0.54	0.25
HBG2	0.40	0.45	0.31	0.39	0.46	0.41	0.36
ATF7	0.63	0.43	0.56	0.32	0.45	0.42	0.36
ATF7IP	0.46	0.46	0.45	0.57	0.58	0.45	0.44
SLC11A2	0.43	0.36	0.46	0.46	0.43	0.35	0.50
KLF5	0.42	0.36	0.50	0.39	0.44	0.48	0.45
HNRNPC	0.42	0.38	0.36	0.36	0.34	0.32	0.27
MTHFD1	0.63	0.39	0.44	0.56	0.56	0.46	0.40
PNP	0.58	0.48	0.50	0.53	0.53	0.36	0.47
APOC1	0.93	0.98	0.77	0.86	0.88	0.84	0.68
APOE	0.95	0.96	0.81	0.89	0.90	0.87	0.72
APOC1P1	0.69	0.60	0.68	0.66	0.61	0.62	0.74
APOC2	0.42	0.40	0.52	0.48	0.53	0.56	0.43
APOC4	0.41	0.42	0.55	0.45	0.46	0.53	0.42
TOMM40	0.91	0.93	0.77	0.78	0.76	0.80	0.71

a. Phenotypes include fluorodeoxyglucose (FDG) and florbetapir (AV45) PET imaging, Functional Activities Questionnaire (FAQ), Clinical Dementia Rating-Sum of Boxes (CDRSB) Alzheimer's Disease Assessment Scale-Cognitive Subscale 13 (ADAS13), Mini-Mental State Examination (MMSE), and Montreal Cognitive Assessment (MoCA).

Supplementary Table S22. Probability of the gene being significantly predictive at 5% level. The pre-specified number of branches for the proposed auto-branch method is four.

Gene	Seven Alzheimer's related phenotypes[a]						
	FDG	AV45	FAQ	CDRSB	ADAS13	MMSE	MoCA
COL11A1	0.55	0.35	0.47	0.52	0.57	0.54	0.54
FCER1G	0.35	0.33	0.35	0.29	0.33	0.38	0.39
GBP2	0.37	0.33	0.50	0.48	0.54	0.41	0.54
HSD11B1	0.36	0.52	0.48	0.40	0.42	0.40	0.49
PARP1	0.50	0.30	0.58	0.51	0.57	0.55	0.53
POU2F1	0.61	0.48	0.42	0.37	0.45	0.50	0.39
NGF	0.41	0.47	0.47	0.50	0.56	0.52	0.55
LHCGR	0.40	0.38	0.33	0.40	0.36	0.37	0.41
LRP2	0.40	0.49	0.39	0.49	0.45	0.52	0.47
APOD	0.46	0.43	0.43	0.42	0.40	0.41	0.44
SST	0.48	0.41	0.50	0.45	0.32	0.37	0.42
ALB	0.51	0.47	0.48	0.40	0.45	0.46	0.36
COL25A1	0.38	0.50	0.41	0.43	0.43	0.47	0.45
ADRB2	0.47	0.37	0.46	0.39	0.47	0.41	0.38
ARSB	0.35	0.38	0.39	0.38	0.35	0.44	0.40
FGF1	0.61	0.51	0.65	0.62	0.62	0.56	0.53
FGF10	0.57	0.44	0.50	0.45	0.46	0.44	0.50
FGF10-AS1	0.47	0.47	0.45	0.39	0.41	0.55	0.49
FGF18	0.44	0.34	0.42	0.56	0.52	0.52	0.41
NDUFS4	0.55	0.62	0.55	0.47	0.35	0.46	0.60
PPP2R2B-IT1	0.40	0.55	0.42	0.45	0.44	0.50	0.46
HSPA1A	0.42	0.45	0.37	0.41	0.49	0.45	0.45
MICA	0.42	0.48	0.40	0.29	0.33	0.45	0.36
MICAL1	0.47	0.51	0.46	0.44	0.65	0.47	0.53
CASC14	0.37	0.41	0.47	0.40	0.39	0.47	0.53
TBP	0.59	0.39	0.48	0.48	0.56	0.46	0.49
TBPL1	0.59	0.51	0.53	0.46	0.49	0.56	0.45
CAV1	0.45	0.41	0.37	0.40	0.42	0.44	0.35
PON3	0.62	0.53	0.55	0.49	0.56	0.43	0.52
RELN	0.59	0.46	0.48	0.57	0.64	0.53	0.65
ADAM9	0.36	0.46	0.40	0.37	0.44	0.41	0.49
NAT1	0.36	0.43	0.45	0.46	0.48	0.51	0.50
NRG1	0.50	0.35	0.43	0.47	0.42	0.33	0.39
DFNB31	0.41	0.36	0.50	0.44	0.53	0.48	0.52
HSPA5	0.50	0.51	0.55	0.46	0.58	0.59	0.56
POMT1	0.58	0.46	0.42	0.48	0.53	0.48	0.43
RXRA	0.39	0.39	0.43	0.46	0.33	0.43	0.27
TLR4	0.28	0.34	0.36	0.51	0.36	0.31	0.44
CACNB2	0.50	0.43	0.38	0.32	0.42	0.46	0.50

MINPP1	0.45	0.48	0.48	0.41	0.45	0.54	0.52
TET1	0.40	0.54	0.51	0.44	0.44	0.46	0.33
APOC3	0.46	0.49	0.43	0.40	0.40	0.51	0.36
HBG2	0.43	0.43	0.34	0.35	0.40	0.50	0.47
ATF7	0.51	0.48	0.52	0.42	0.51	0.40	0.39
ATF7IP	0.52	0.43	0.50	0.53	0.60	0.42	0.40
SLC11A2	0.41	0.47	0.41	0.43	0.49	0.39	0.46
KLF5	0.51	0.49	0.49	0.38	0.49	0.43	0.39
HNRNPC	0.44	0.39	0.44	0.37	0.33	0.43	0.38
MTHFD1	0.61	0.37	0.37	0.53	0.51	0.47	0.48
PNP	0.69	0.53	0.50	0.53	0.53	0.40	0.47
APOC1	0.89	0.96	0.75	0.87	0.87	0.84	0.70
APOE	0.90	0.95	0.83	0.84	0.89	0.90	0.77
APOC1P1	0.67	0.57	0.75	0.60	0.65	0.63	0.65
APOC2	0.41	0.41	0.48	0.47	0.51	0.46	0.46
APOC4	0.39	0.36	0.46	0.44	0.49	0.42	0.49
TOMM40	0.86	0.93	0.73	0.78	0.78	0.78	0.70

a. Phenotypes include fluorodeoxyglucose (FDG) and florbetapir (AV45) PET imaging, Functional Activities Questionnaire (FAQ), Clinical Dementia Rating-Sum of Boxes (CDRSB) Alzheimer's Disease Assessment Scale-Cognitive Subscale 13 (ADAS13), Mini-Mental State Examination (MMSE), and Montreal Cognitive Assessment (MoCA).

Supplementary Table S23. Average Pearson correlations for seven traits under the settings of simulation 1.

Sharing Situations	Multi-Lasso	HPS[a]	2 Groups HPS[b]	3 Groups HPS[c]	4 Groups HPS[d]	STL[e]
		$\sigma_\beta/\sigma_\epsilon$ = 1:3				
Complete Sharing	0.463	0.531	0.503	0.484	0.474	0.433
Two-group Sharing	0.425	0.478	0.477	0.472	0.458	0.377
Three-group Sharing	0.404	0.420	0.433	0.432	0.426	0.416
Four-group Sharing	0.402	0.402	0.428	0.419	0.423	0.398
No Sharing	0.393	0.322	0.379	0.395	0.402	0.395
		$\sigma_\beta/\sigma_\epsilon$ = 1:4				
Complete Sharing	0.349	0.438	0.404	0.386	0.368	0.343
Two-group Sharing	0.313	0.377	0.370	0.364	0.349	0.319
Three-group Sharing	0.291	0.320	0.324	0.327	0.323	0.290
Four-group Sharing	0.293	0.294	0.314	0.314	0.314	0.300
No Sharing	0.289	0.220	0.280	0.282	0.302	0.291
		$\sigma_\beta/\sigma_\epsilon$ = 1:5				
Complete Sharing	0.266	0.356	0.321	0.305	0.294	0.259
Two-group Sharing	0.239	0.274	0.284	0.279	0.273	0.254
Three-group Sharing	0.215	0.218	0.240	0.234	0.234	0.229
Four-group Sharing	0.220	0.199	0.231	0.236	0.232	0.230
No Sharing	0.219	0.140	0.195	0.202	0.218	0.219
		$\sigma_\beta/\sigma_\epsilon$ = 1:6				
Complete Sharing	0.207	0.290	0.250	0.249	0.222	0.210
Two-group Sharing	0.183	0.218	0.221	0.208	0.202	0.186
Three-group Sharing	0.163	0.153	0.173	0.174	0.177	0.174
Four-group Sharing	0.169	0.129	0.161	0.170	0.179	0.173
No Sharing	0.171	0.078	0.136	0.145	0.159	0.169

a. Hard parameter sharing across all layers except the last one.
b. The pre-specified number of branches for the auto-branch is two and traits in different branches are trained using separate hard parameter sharing model.
c. The pre-specified number of branches for the auto-branch is three and traits in different branches are trained using separate hard parameter sharing model.
d. The pre-specified number of branches for the auto-branch is four and traits in different branches are trained using separate hard parameter sharing model.
e. Each trait is modeled independently without accounting for trait correlations.

Supplementary Table S24. Average Pearson correlations for seven traits under the settings of simulation 2.

$\sigma_\beta^2/\sigma_{\beta_s}^2$	Multi-Lasso	HPS[a]	2 Groups HPS[b]	3 Groups HPS[c]	4 Groups HPS[d]	STL[e]
		$(\sigma_\beta^2+\sigma_{\beta_s}^2)/\sigma_\epsilon^2$ = 1:9				
1:9	0.444	0.495	0.468	0.453	0.449	0.416
3:7	0.440	0.428	0.442	0.432	0.434	0.411
5:5	0.429	0.404	0.425	0.426	0.427	0.400
7:3	0.350	0.390	0.424	0.429	0.428	0.395
9:1	0.357	0.329	0.380	0.394	0.403	0.369
		$(\sigma_\beta^2+\sigma_{\beta_s}^2)/\sigma_\epsilon^2$ = 1:16				
1:9	0.324	0.397	0.370	0.362	0.352	0.326
3:7	0.306	0.335	0.331	0.327	0.323	0.297
5:5	0.314	0.294	0.315	0.311	0.311	0.295
7:3	0.299	0.245	0.278	0.282	0.284	0.280
9:1	0.299	0.234	0.284	0.288	0.293	0.273
		$(\sigma_\beta^2+\sigma_{\beta_s}^2)/\sigma_\epsilon^2$ = 1:25				
1:9	0.215	0.271	0.253	0.247	0.233	0.232
3:7	0.228	0.268	0.259	0.250	0.242	0.222
5:5	0.217	0.223	0.226	0.228	0.229	0.224
7:3	0.214	0.151	0.191	0.198	0.200	0.203
9:1	0.216	0.134	0.190	0.194	0.206	0.215
		$(\sigma_\beta^2+\sigma_{\beta_s}^2)/\sigma_\epsilon^2$ = 1:36				
1:9	0.201	0.259	0.224	0.215	0.209	0.200
3:7	0.187	0.212	0.194	0.190	0.196	0.177
5:5	0.176	0.152	0.166	0.167	0.163	0.170
7:3	0.187	0.131	0.164	0.165	0.169	0.177
9:1	0.168	0.086	0.124	0.144	0.148	0.155

a. Hard parameter sharing across all layers except the last one.
b. The pre-specified number of branches for the auto-branch is two and traits in different branches are trained using separate hard parameter sharing model.
c. The pre-specified number of branches for the auto-branch is three and traits in different branches are trained using separate hard parameter sharing model.
d. The pre-specified number of branches for the auto-branch is four and traits in different branches are trained using separate hard parameter sharing model.
e. Each trait is modeled independently without accounting for trait correlations.
